# A Refractive Index Study of a Diverse Set of Polymeric Materials by QSPR with Quantum-Chemical and Additive Descriptors

**DOI:** 10.3390/molecules25173772

**Published:** 2020-08-19

**Authors:** Meade E. Erickson, Marvellous Ngongang, Bakhtiyor Rasulev

**Affiliations:** Department of Coatings and Polymeric Materials, North Dakota State University, Fargo, ND 58108, USA; meade.erickson@ndsu.edu (M.E.E.); marvellous.ngangong@ndsu.edu (M.N.)

**Keywords:** refractive index, polymers, descriptors, QSAR, QSPR, polarizability, ionization potential

## Abstract

Predicting the activities and properties of materials via in silico methods has been shown to be a cost- and time-effective way of aiding chemists in synthesizing materials with desired properties. Refractive index (n) is one of the most important defining characteristics of an optical material. Presented in this work is a quantitative structure–property relationship (QSPR) model that was developed to predict the refractive index for a diverse set of polymers. A number of models were created, where a four-variable model showed the best predictive performance with R^2^ = 0.904 and Q^2^_LOO_ = 0.897. The robustness and predictability of the best model was validated using the leave-one-out technique, external set and y-scrambling methods. The predictive ability of the model was confirmed with the external set, showing the R^2^_ext_ = 0.880. For the refractive index, the ionization potential, polarizability, 2D and 3D geometrical descriptors were the most influential properties. The developed model was transparent and mechanistically explainable and can be used in the prediction of the refractive index for new and untested polymers.

## 1. Introduction

The refractive index is an important property of polymers in optical applications due to the property defining the velocity at which light travels through the material relative to a vacuum [1,2,3,4,5,6,7,8]. The refractive index has been widely known since before the 19th century, and has been used to understand the optical activities of many materials [9].

There are many uses polymers have in industry today. High refractive index polymers (HRIP) have been studied by many for industrial process applications [5,10,11,12]. Some of the applications include complementary metal oxide semiconductor image sensory (CIS) [13], polymer films for high-performance antireflection coatings [14], UV nanoimprinting lithography [15], the covering of light-emitting diodes [16], and coatings of fiber gratings [17]. The uses that are shown were recently found and many more niches will be found in the future.

Recent works that investigated refractive index refer to using a refractometer which correlates the amount that light changes direction when passing through a substance [18,19,20]. The Standard Test Method for Index of Refraction of Transparent Organic Plastics, ASTM D542, has been used by some researchers [18]. Crena et al. used a refractometer to measure the refractive index of mixtures of styrene–methyl methacrylate and glycidyl methacrylate. It was reported that the desired range of refractive index of 1.5750–1.5894 was affected by the different mixtures of styrene–methyl methacrylate–glycidyl methacrylate. Others such as Kasaroa et al. used similar methods when specifying the different wavelengths, or colors, to determine the change in the refractive index of poly methyl methacrylate, polystyrene, and others [20]. Another method has been suggested recently, that of using a scanning angle Raman spectroscopy for nondestructive testing [21]. This method uses a laser pointed toward a sapphire prison which is focused onto the imaging spectrometer to which the refractive index can be translated from the information gathered, and was found to find values within 10% comparing to other methods. These methods use different strategies in obtaining the refractive index of polymers.

Differing techniques have been used to model the refractive index of differing materials such as using the Lorentz–Lorenz equation, Mie Theory, the Rayleigh–Debye–Gans theory, the implementation of group contribution method, and the use of quantitative structure property relationship (QSPR) methodology [22,23]. QSPR has been used as a powerful tool to predict the properties of various chemical systems and materials for the last three decades [22,23,24,25,26,27,28,29,30]. It is worth noting that an interesting approach to predict the refractive index for aerosols can be applicable for polymers, which is represented in the work [29]. The earliest modeling of the refractive index of polymers using QSPR methods concluded that quantum-chemical descriptors such as HOMO–LUMO gap and nuclear repulsion for C–H bond heavily affected the refractive index [31]. This five-variable model was found to have *R^2^* = 0.940, *F* = 282.13, and *S^2^* of 3.13 × 10^−4^ with an average prediction error of 0.9%. This model only used 655 descriptors and 95 amorphous polymers, where more are available today [31]. A later model was created by Xu et al. by creating a dataset of 121 linear polymers [32]. The four variable model included: the sum of valence degrees, the degree of unsaturation, the relative number of halogen atoms, and the electrostatic attraction between the main chains. The *R^2^* and prediction error were found to be 0.964 and 0.87% respectively [32]. 

Jabeen et al. published a model using a dataset of 127 diverse polymers, which achieved values of *R^2^* of 0.932, *R^2^_ext_* of 0.882, *Q^2^_LOO_* of 0.922, and *Q^2^-F1* of 0.875. The four-variable model asserted that polarizability, sp^2^ hybridization and the frequency of C–F at distance 1 affected the refractive index greatly [33]. A set of models that Khan et al. presented used only 2D descriptors and had 221 polymers in the dataset. These six-variable models were created by partial least squares (PLS) and were shown for comparison. All four models included the descriptor of MLFER_E, which is the molecular linear free energy relation and relates polarizability and the solute/solvent interaction through n- and pi-electron pairs. Moreover, the mean ionization potential descriptor (Mi) was in each model. Each of the four models had at least three latent variables and ranged from 0.899 to 0.895 for the *R^2^* value [34]. These descriptors that relate Mi and the interaction of electron pairs are a large factor in many of these models. Duchowicz et al. developed a model using QSPR methods with a dataset of 234 structurally diverse polymers [35]. The model considered using 1–5 monomeric repeating units to generate flexible descriptor models. The paper describes a descriptor model of linear combination of correlation weights (DCW_4_) that used to model the refractive index of polymers. It is worth noting that the DCW descriptor is actually a latent variable and combines a number of descriptors in it, so it is not a single descriptor. The statistical values of R^2^ of 0.96 and R^2^_ext_ 0.85 were reported for this model.

The current work discusses a newly developed model to predict a refractive index of the diverse set of polymers. A large set of 262 polymers was used and collected from multiple sources [1,2,3,4,6,7], and the collected set was larger than in previously reported works [31,32,33,34,35]. The current work resulted in the development of a robust four-variable model. The applied descriptors included several categories, including topological, quantum-chemical, functional group counts, constitutional, geometrical, and topological. The developed model has encompassed the combination of the largest number of polymers and the largest number of an initial set of descriptors to generate a robust, transparent and mechanistically explainable model. The generated model was internally and externally validated by different techniques such as the leave-one-out technique, y-scrambling, *r^2^_m_ave_* , *CCCcv* metrics, and splitting the data into prediction and training sets.

## 2. Materials and Methods

### 2.1. Collection and Preparation of Experimental Dataset

A diverse dataset of 262 polymers was collected from multiple sources [1,2,3,4,6,7,35]. The dataset included, organic-based polymers such as cellulose acetate and non-renewable-sourced polymers such as poly(ethylene). This dataset includes polyamides, polyesters, polyolefins, polysilylenes and others. Information on the collected data of polymers is shown in the Supplementary Information in Tables S1 and S2 [1,2,3,4,6,7,35]. Table S1 shows the structure and number it was assigned to in the dataset. Table S2 includes the SMILES (Simplified Molecular-Input Line-Entry System) notations for monomers, the chemical names produced by ChemDraw 16 [36], the experimental refractive index, the logarithmically transformed experimental refractive index, the predicted logarithm refractive index, and which set the specific compounds were a part of. Any duplicates and unknown polymers/polymer mixtures were removed. Each polymer was drawn in a polymerized monomer 2D structure format using ChemDraw 16 [36]. These monomer structures were “end-capped” with hydrogen atoms for consistent monomer functionality. This monomer end-capping format was implemented due to the limitations of a computational descriptor generation of long polymer chains. The end-capping technique was done to replicate the monomer structure functionality after being polymerized. 

The monomer structures were then optimized using HyperChem 8 [37]. The dataset was then split into training and prediction sets, approximately 75% of the 262 were used as the training set and the other 25% were used as the prediction set. The training and prediction sets were used for model generation and validation, respectively. The refractive index value was then converted to a logarithmic scale for convenient comparing the results to previously published works and for the linearity of the data to the free energies.

### 2.2. Descriptors Set Generation

The monomer unit was drawn using ChemDraw 16 [36]. Each monomer structure was optimized in HyperChem 8 [37], using the MM+ force-field. Then, a set of quantum descriptors was calculated, including the highest occupied molecular orbital (HOMO), the lowest unoccupied molecular orbital (LUMO), the dipole moment, the energy gap between HOMO and LUMO, and the ionization potential. These descriptors were calculated using a semi-empirical method RM1 [37]. The rest of the descriptors were generated using Dragon 6 [38] by inputting in the structures optimized in HyperChem. Dragon 6 generated about 4500 descriptors per structure [38]. These descriptors include the following categories: constitutional indices, 2D and 3D matrix-based descriptors, 2D autocorrelations, topological descriptors, charge-based descriptors, 0D, 2D, and 3D descriptors, molecular properties and more. Descriptors with high correlation, single variables, and non-informative information were discarded based on constant value, near constant, and pair correlation criteria.

### 2.3. Model Development and Validation

For the QSPR correlation, a combined genetic algorithm (GA) and multi-linear regression analysis (MLRA) method was used to develop the models for this work. The QSARINS software [39] was used for the final steps in the models’ development. The dataset was accompanied by the log(n) value of the refractive index associated with each polymer structure for model development. The training set and prediction sets were created by sorting tool in QSARINS, and every fourth structure (25%) was assigned to the prediction set in order of ascending refractive index value. The dataset was split into the training set with 203 structures and the prediction set with 66 structures. The descriptors chosen for the model were determined by genetic algorithm (GA).

A particular setup during the model development was used to select a best model. Thus, the number of generations was set to 2000, the population size of the final model was set to 20, and a mutation rate of 40% was used. For validation purposes, multiple methods were applied, including leave-one-out (LOO) cross validation, leave-many-out (LMO), y-scrambling, as well as internal and external validation protocols. Some of these methods were used to show the possible existence of fortuitous correlations. After validation techniques were applied, the best model was chosen based on multiple criteria: (1) high statistical performance variables such as R^2^ and Q^2^ (including R^2^-Q^2^ < 0.3); (2) lowest number of outliers; (3) a low number of variables in the model; and (4) low cross-correlation between descriptors in the selected model.

## 3. Results and Discussion

**Figure 1:**
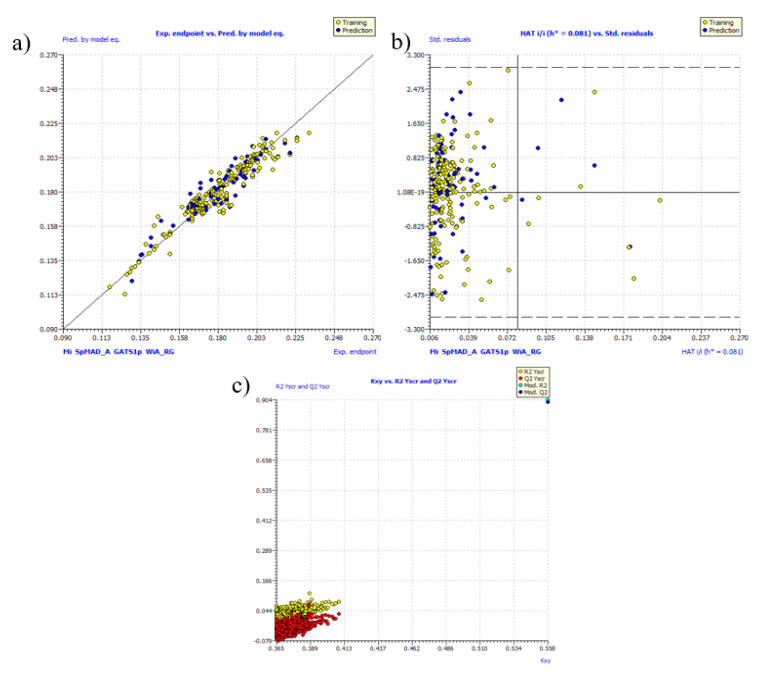
(**a**) Correlation plot of the experimental refractive index data vs. that predicted by the model; (**b**) Williams plot; and (**c**) the *y*-scrambling plot, where the red dots are for the *R^2^* values and the yellow dots are for *Q^2^* values.

The molecular structures and descriptor information, together with refractive index values were analyzed by QSARINS [39], applying the GA-MLR technique. Numerous models were generated with one to five descriptors in the model. An initial developmental run of models was executed with the most notable models with high statistical values. A developed Williams plot for a best four-variable model in shown in Figure 1, with all points shown within the three standard deviation limits within the applicability domain (AD). The AD was developed using the leverage approach [40] 

The results of the best QSPR model for models with from 1 to 5 descriptors are shown in Table 1, where the performance data are shown for the training and prediction sets. In Table 1, the following performance indicators are shown: *R^2^* is the regression coefficient for the training set, *R^2^_adj_* is an adjusted *R^2^*, *S* represents the average distance the observed values are away from the regression line, *Q^2^_LOO_* is the “leave-one-out” coefficient, *CCCcv* is “concordance correlation coefficient cross-validation”, *R^2^y-scr* and *Q^2^y-scr* are for the “y-scrambling” performance coefficients, *RMSE_tr_* is a “root-mean-square error”, and *r^2^_m_ave_* is the “metrics parameters average” that shows the robustness of the model. These are all internal and cross-validation parameters which test the predictive ability and robustness of the model.

Concordance correlation coefficient (CCC) was calculated as a more restrictive parameter for expressing the external predictivity of each model, as shown in Equation (1) [41]:

(1) $$CCC=\frac{2\sum_{j=1}^{\mathrm{kEXT}}\left(y_{j}^{\mathrm{obs}}-{\overset{´}{y}}^{\mathrm{obs}}\right)\left(y_{j}^{\mathrm{pred}}-{\overset{´}{y}}^{\mathrm{pred}}\right)}{\sum_{j=1}^{\mathrm{kEXT}}{\left(y_{j}^{\mathrm{obs}}-{\overset{´}{y}}^{\mathrm{obs}}\right)}^{2}+\sum_{j=1}^{\mathrm{kEXT}}{\left(y_{j}^{\mathrm{pred}}-{\overset{´}{y}}^{\mathrm{pred}}\right)}^{2}+k_{\mathrm{EXT}}{\left(y_{j}^{\mathrm{obs}}-{\overset{´}{y}}^{\mathrm{pred}}\right)}^{2}}$$

The variables for Equation (1) are as follows: $y_{j}^{\mathrm{obs}}$ is the experimental (observed) value of the property for the i^th^/j^th^ compound; $y_{j}^{\mathrm{pred}}$ is the predicted value for i^th^/j^th^ compound; $\tilde{y}$ is the mean experimental value of the property in the training set while $\hat{y}$ is the mean experimental value of the validation set; *k* is the number of compounds in the validation set.

The fourth variable model was chosen as the best model due to its high *R^2^* value; its passing internal, external, and validation criteria. The descriptors of the chosen model and their statistical coefficients are shown in Table 2. First involved descriptor is a Mi descriptor, which is a mean first ionization potential generated by the Dragon software [38]. The ionization potential is the amount of energy required to remove an electron from a gaseous atom or ion. The ionization potential specifies the energy required for the first excited electron to leave. According to Koopman′s theory, ionization energy is the negative value of HOMO energy [42]. In addition, Reddy et al. had shown in experimental work that ionization potential negatively influenced the refractive index while working with alkali halides [43]. Second descriptor, GATS1p is a descriptor weighted by polarizability [38], Table 2. Polarizability is the ability, of an atom or molecule, to form instantaneous dipole in reaction to an external field i.e., magnetic or electrical. Polarizability is related to the refractive index via the Lorentz–Lorenz expression [44,45]. 

Specifically, regarding the descriptor of GATS1p, it is actually the Geary coefficient which is weighted by polarizability. When weighing data it is known the weighing factor, in this case polarizability, decreases the descriptor with the increase in weighing factor. This relates back to the model by suggesting that as polarizability increases, so does the refractive index, but due to polarizability being a weighing factor, the GATS1p descriptor negatively affects the refractive index which matches the model.

The two discussed descriptors (Mi, GATS1p) are related to the excitation energies of ground state electrons [43,46]. For example, optical polarizability in quantum theory results from a mixing of suitable excited state wave functions with the ground state wave function. The mixing coefficient is inversely proportional to the excitation energy from the ground to the excited state. A small HOMO–LUMO gap (and a higher energy of HOMO and a lower ionization potential) automatically means small excitation energies to the manifold of excited states. Therefore, soft molecules, with a small gap, will be more polarizable than hard molecules. Based on the QSPR model obtained, these two descriptors have been found to heavily influence the refractive index due to their interaction with light that passes through the materials.

Polymer properties are known to be affected by the chemical structure of the monomer units, as well as the bulk interactions of the chains [47]. In the developed model, the monomer unit length is represented by WIA_RG and SpMAD_A descriptors, which are 2D and 3D matrix descriptors. The size and length of a molecule affect the speed of light by creating a “barrier” of material that light needs to travel through. The length of polymeric units/chains (monomers) has also been expressed to increase the refractive index of the material with branching [48]. It is believed that the larger the molecule, the larger the refractive index which is due to the previously mentioned material “barrier.”

The Figure 1 represents the plots of experimental and predicted data correlation (a), Williams plot (b) and the y-scrambling analysis plot (c). Thus, Figure 1a is the correlation plot for the training (yellow dots) and the prediction sets (blue dots). The represented plot for the best 4-variable model has an *R^2^* value of 0.904 and a very good *Q^2^_LOO_* of 0.897. With these values being high and comparable, the internal validation considers the model to be stable and internally robust. Figure 1b shows the Williams plot and all values are within three standard deviations. The molecules that are outside the *HAT* values are due to structural differences only, and are within the applicability domain. The y-scrambling plot is generated and represented in Figure 1c to ensure that the selected model was not chosen by chance. Due to the model showing compliance with the applied validation, the model is concluded to be highly predictive and robust.

The Williams plot allows to evaluate the deviations from the ideal matching between experimental and predicted data. Figure 1b shows that there are no anomalous trends, which is evident from the regular distribution of points in both halves of the plot. The twelve structures which are out of the applicability domain (with the *HAT* value larger than 0.075) are not outliers, they represent structurally peculiar compounds. These twelve structures (1, 2, 3, 17, 21, 30, 38, 85, 125, 166, 235, 237) have log(n) values of refractive indexes that range within 0.117–0.204, well in the range of all other data points. 

It is necessary to iterate the robustness criteria for internal and external validation tests. In our case, the chosen model has values of 0.904 and 0.897 for *R^2^* and *Q^2^_LOO_*, respectively, which are the results from the internal validation tests. These values are shown in Table 2. To ensure predictability, the external validation was conducted based on the external test set, *R^2^_ext_*. With the current model, the *R^2^_ext_* was found to be 0.880. This was acceptable for an external validation test and reinforces the predictive ability of the model. Further criteria were used to ensure the external predictive capability by the predictive squared correlation coefficients of *Q^2^-F1, Q^2^-F2, Q^2^-F3*. The coefficient values for the chosen model were 0.874, 0.873, and 0.899, respectively, which confirm once again the high predictive performance of the selected model. Other works have shown that the concordance correlation coefficient cross-validation (*CCCcv*) and metrics parameters (*r^2^_m_ave_*) can be used for additional validation. The chosen model has a *CCCcv* of 0.946. This further reinforces the stability and predictive ability of the developed model. Moreover, the *r^2^_m_ave_* average was found to be 0.824 which adds to the predictive capability. The root means square error (*RMSE*) has low values of 0.007 and 0.008 for internal and external validation. 

With all the internal and external criteria being surpassed by the model, it is confirmed that the model is robust, stable and can be used to predict the refractive index of polymers. It has been validated by passing *R^2^* and *Q^2^_LOO_*, *RMSE*, internal and external *CCC* and *r^2^_m_ave_* for training and prediction sets. Additionally, the y-scrambling validation procedure is confirmed the robustness and no chance correlation. 

## 4. Conclusions

A four-variable QSPR model was developed using a comprehensive and diverse dataset of 262 polymers. The influential descriptors in the model were found to be the ionization potential (Mi), the descriptor weighted by polarizability (GATS1p), and the molecular structural topology (WiA_RG and SpMAD_A). This model was found to have an *R^2^* value of 0.904 while the internal and external validation parameters were found to be *Q^2^_LOO_* of 0.897, and an *R^2^_ext_* of 0.880. The model was also subjected to and passed the y-scrambling, *CCCcv, R^2^_m_ave_* and applicability domain examinations.

The ionization potential (Mi) and polarizability (GATS1p) were connected to the refractive index via excitation energies of ground state electrons. These descriptors have a large influence on the refractive index due to their interaction with the energy gap of ground state and excited state electrons. The matrix descriptors of WiA_RG and SpMAD_A are associated with the molecular structural topology and size. By varying the shape and size of the molecule, light has a different path to go through which varies the speed of light and changes the refractive index. 

The characteristics of this model as well as the internal and external validation parameters confirm the predictive ability and robustness of this model. Further polymers with optical uses can be developed using this transparent and reproducible model for the development of high or low refractive indices. 

## Figures and Tables

**Table 1:** List of the best models with 1–5 variables.

Variable Number	Equations	$R^{2}$	$R_{adj}^{2}$	$Q_{Loo}^{2}$	RMSE_tr_	r^2^_m_ave_	R^2^_ext_	RMSE_ext_	CCCcv
1	Log(Ri) = −0.1124 Mi + 0.205	0.676	0.675	0.665	0.012	0.559	0.684	0.010	0.802
2	Log(Ri) = −0.080 Mi – 0.049 GATS1p + 0.222	0.847	0.845	0.840	0.008	0.752	0.867	0.009	0.913
3	Log(Ri) = –0.046 GATS1p – 0.029 VR2_G – 0.064 R2s +0.228	0.883	0.881	0.876	0.007	0.847	0.921	0.008	0.934
4	Log(Ri) = −0.068 Mi + 0.031 SpMAD_A – 0.048 GATS1p + 0.040 WiA_RG + 0.191	0.904	0.902	0.897	0.007	0.842	0.880	0.008	0.946
5	Log(Ri) = –0.017 SpMAD_D/DT – 0.039 GATS1p – 0.019 VR2_G + 0.009 G2m – 0.075 H0i + 0.238	0.907	0.903	0.899	0.006	0.886	0.823	0.009	0.938

**Table 2:** Description and statistical coefficients of the descriptors in the 4-variable model.

Name of Descriptor	Coefficient	Std. Coeff.	Co. Int. 95%	Description	Type of Descriptor
Intercept	0.191	------	0.009	------	------
Mi	−0.068	−0.491	0.009	mean first ionization potential (scaled on Carbon atom)	Constitutional indices
GATS1p	−0.048	−0.443	0.006	Geary autocorrelation of lag 1 weighted by polarizability	2D autocorrelations
WiA_RG	0.040	0.266	0.009	average Wiener-like index from reciprocal squared geometrical matrix	3D matrix-based descriptors
SpMAD_A	0.031	0.281	0.009	spectral mean absolute deviation from adjacency matrix	2D matrix-based descriptors

## Supplementary Materials

The Supplementary Information file is available online alongside the manuscript.
