# Supplementary material for: A Refractive Index Study of a Diverse Set of Polymeric Materials by QSPR with Quantum-Chemical and Additive Descriptors
Source: Molecules. 2020 Aug 19;25(17):3772. doi: 10.3390/molecules25173772 (PMC7503810; doi:10.3390/molecules25173772)
Supplement: Supplementary file 1 [file molecules-25-03772-s001.pdf]

Supporting Information

A Refractive Index Study of a Diverse Set of Polymeric Materials by QSPR with quantum-chemical and additive descriptors

Meade E. Erickson, Marvellous Ngangong, Bakhtiyor Rasulev*

Department of Coatings and Polymeric Materials, North Dakota State University, Fargo, ND 58108

*Corresponding author: B.R. email: bakhtiyor.rasulev@ndsu.edu

Table S1

Number	Structure
1	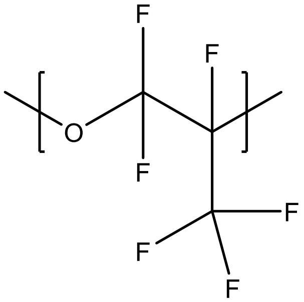

2	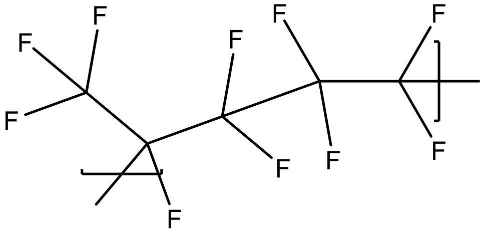
3	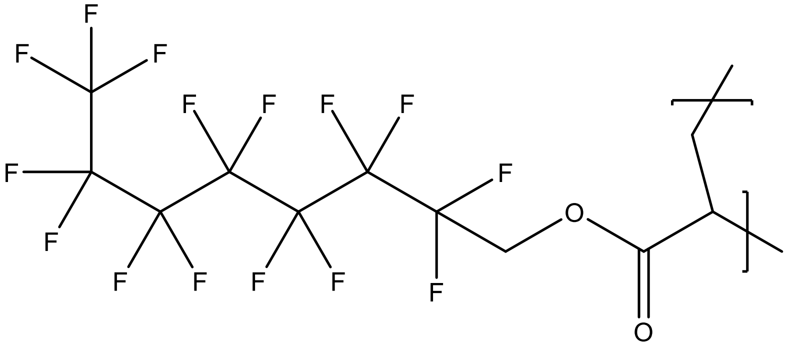
4	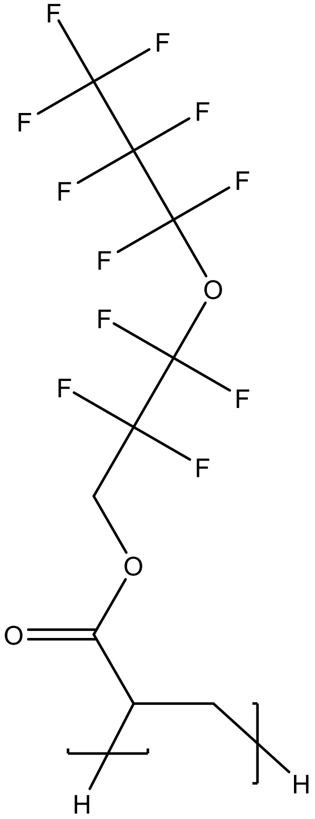

5	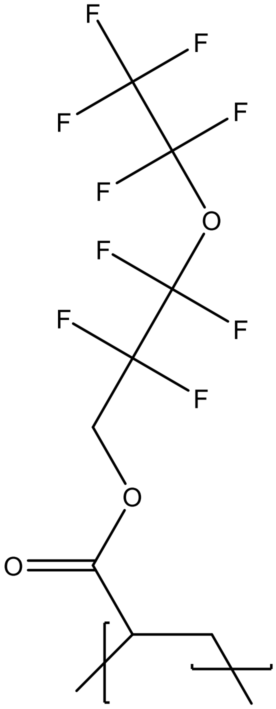
6	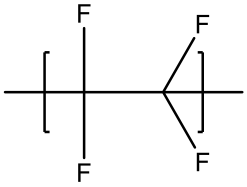
7	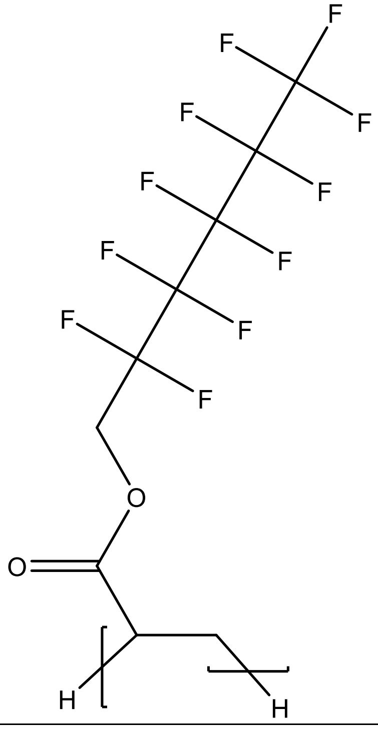

8	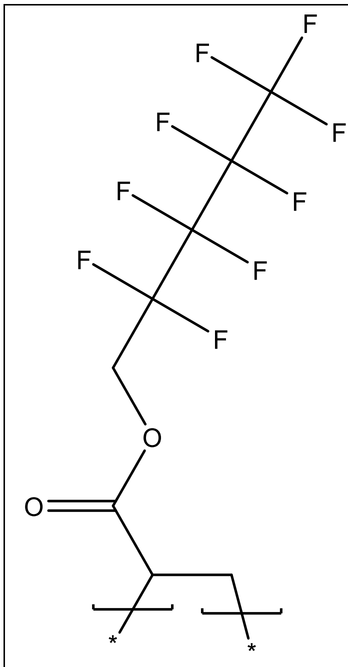
9	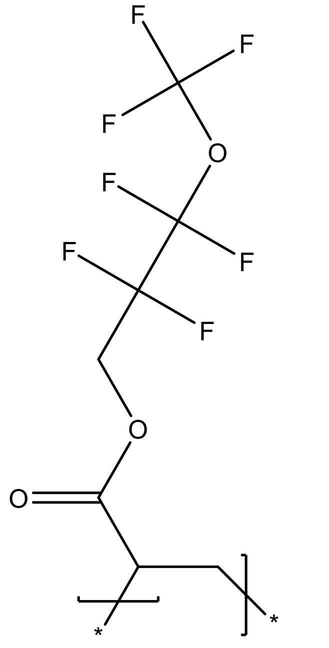

10	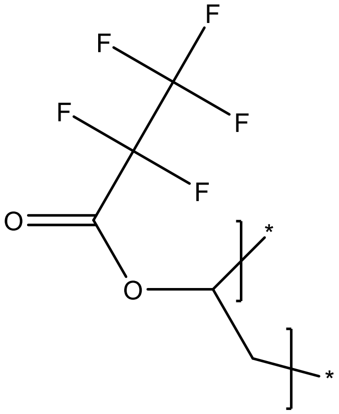
11	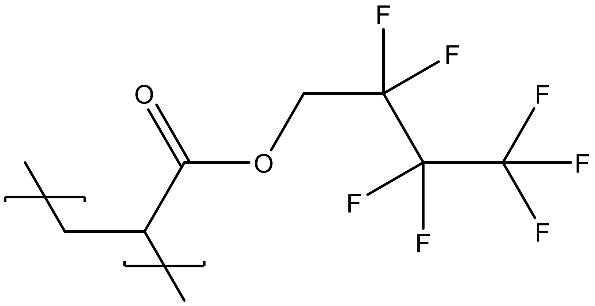
12	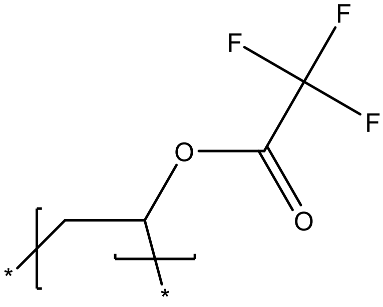
13	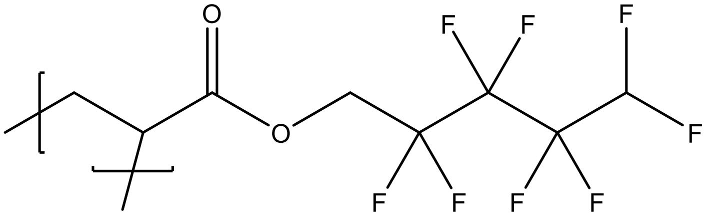
14	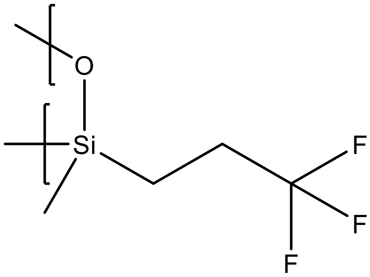

15	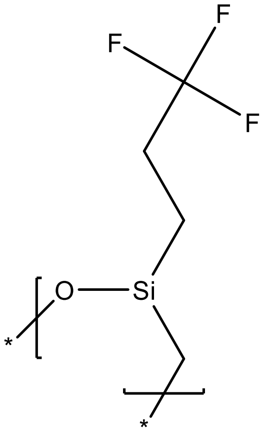
16	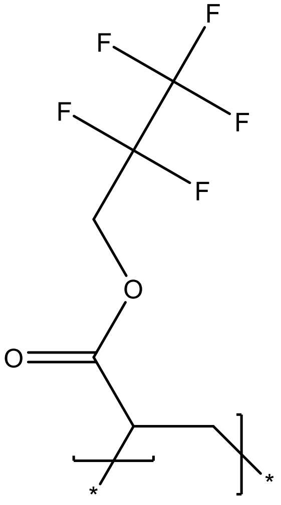
17	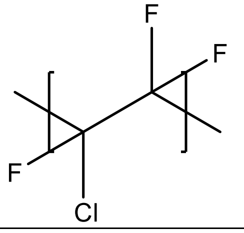

18	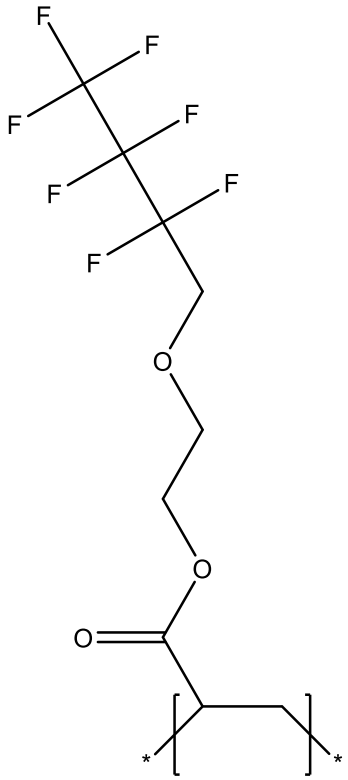
19	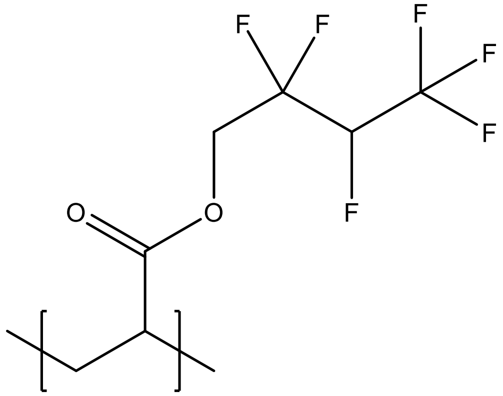
20	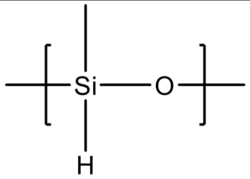
21	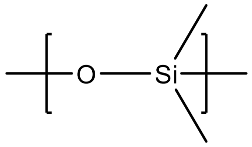

22	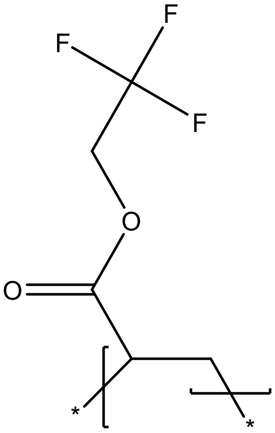
23	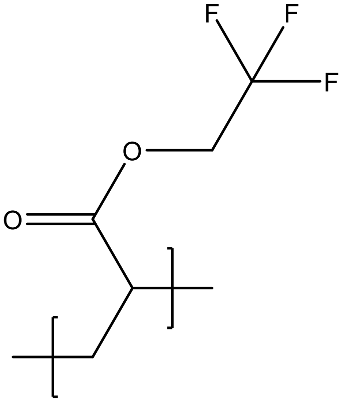
24	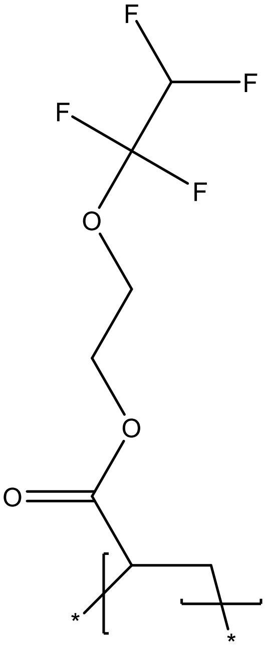

25	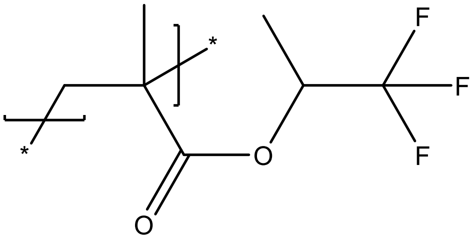O<br>O<br>F<br>F<br>F
26	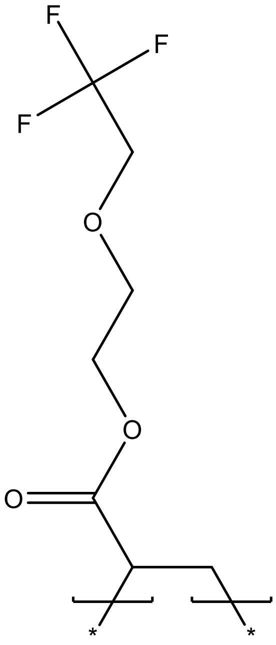F<br>F<br>F<br>O<br>O<br>O
27	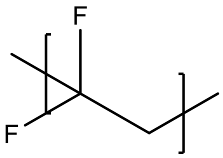F<br>F
28	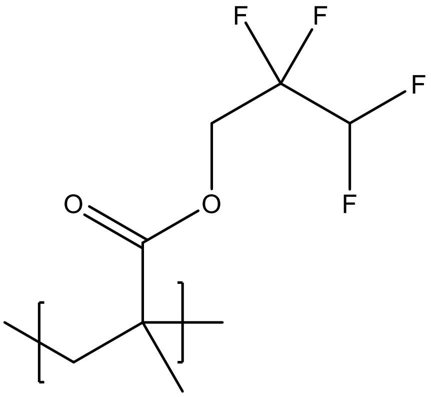F<br>F<br>F<br>F<br>O<br>O
29	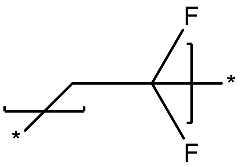F<br>F

30	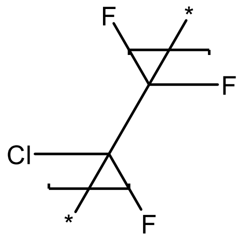
31	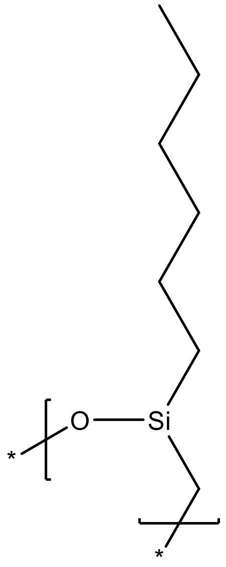

32	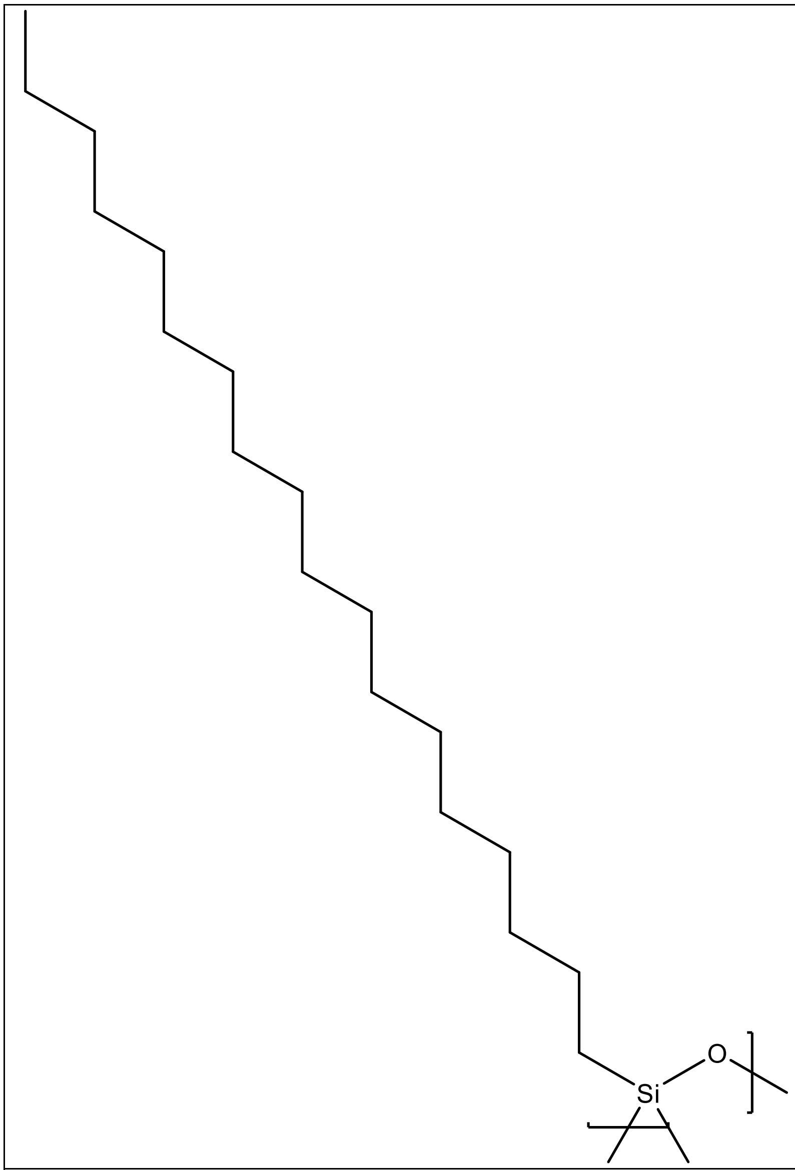
33	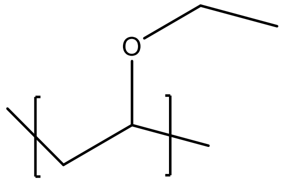
34	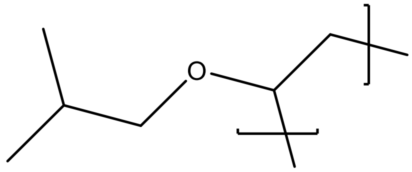
35	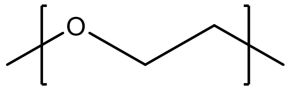

36	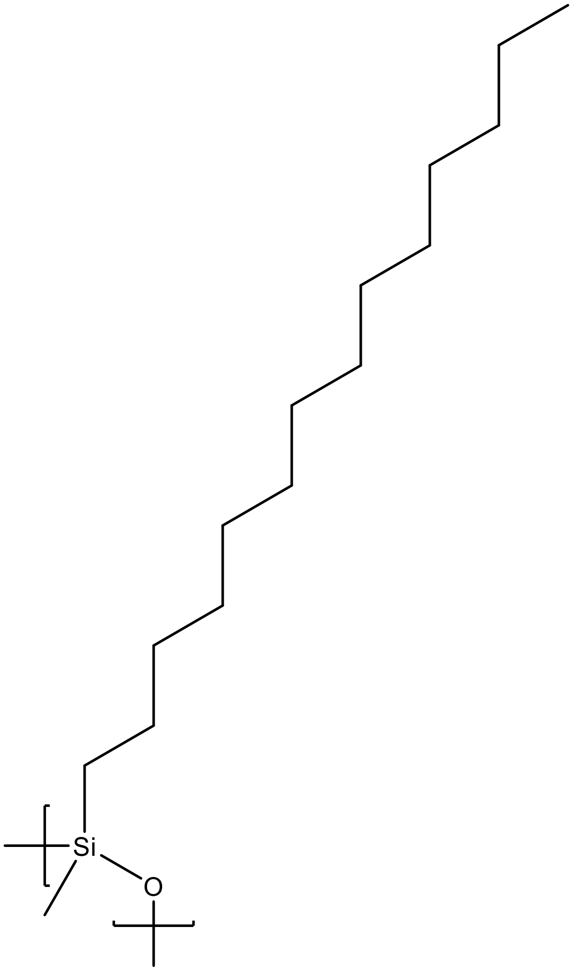
37	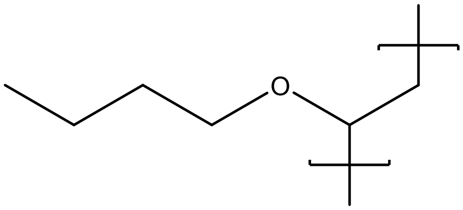
38	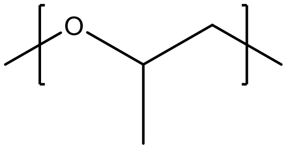

39	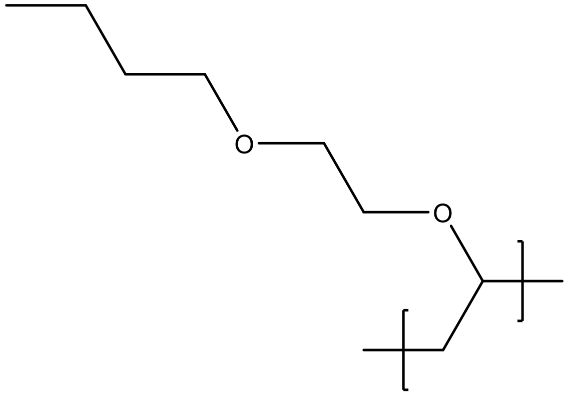
40	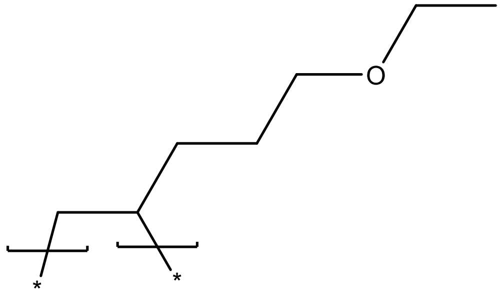
41	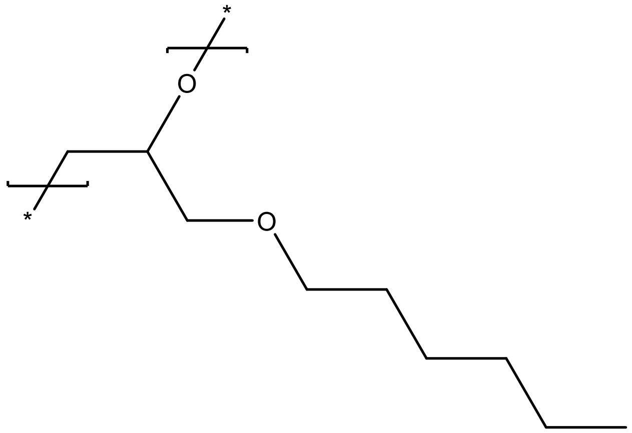
42	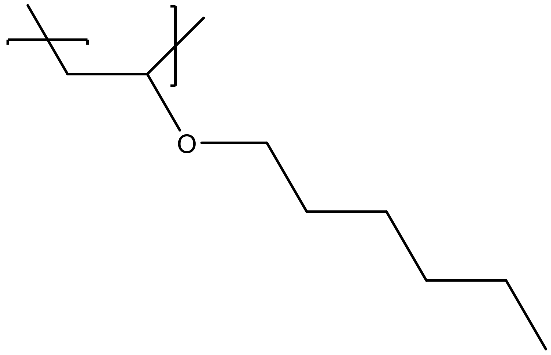

43	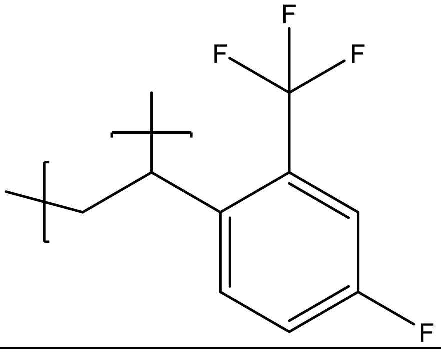
44	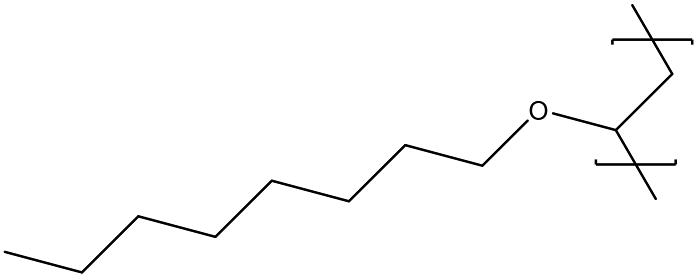
45	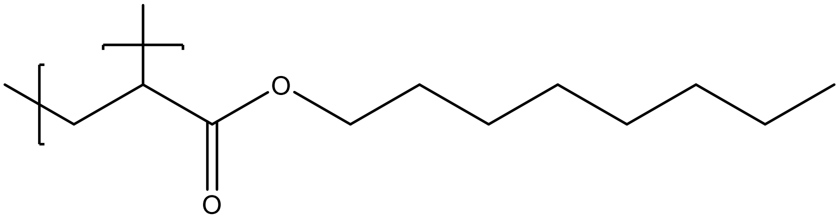
46	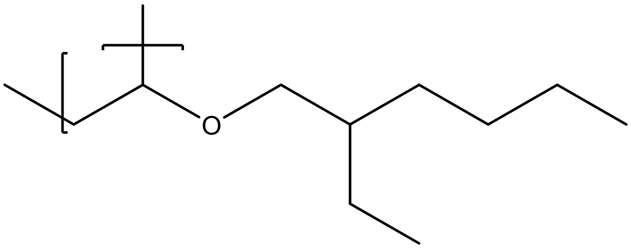
47	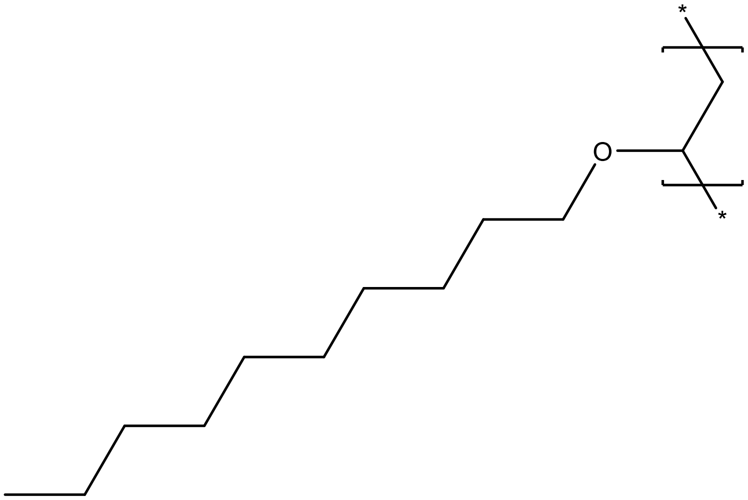

48	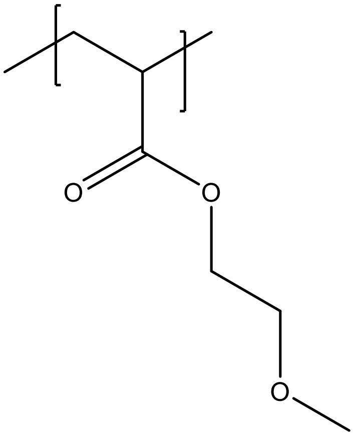
49	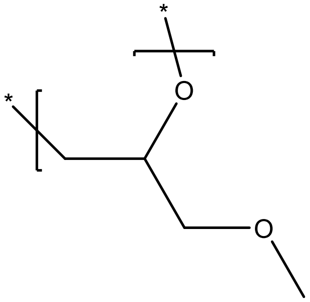
50	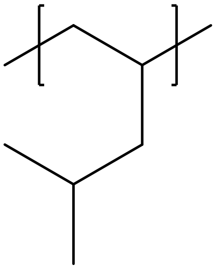
51	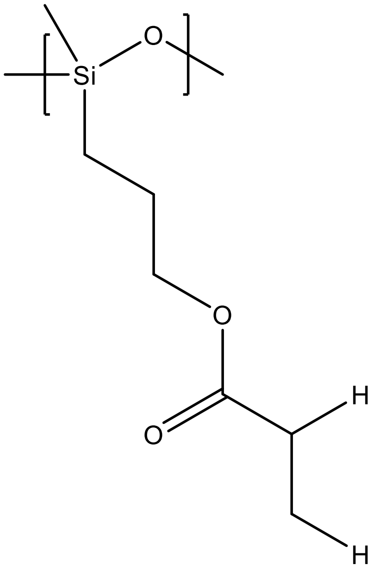

52	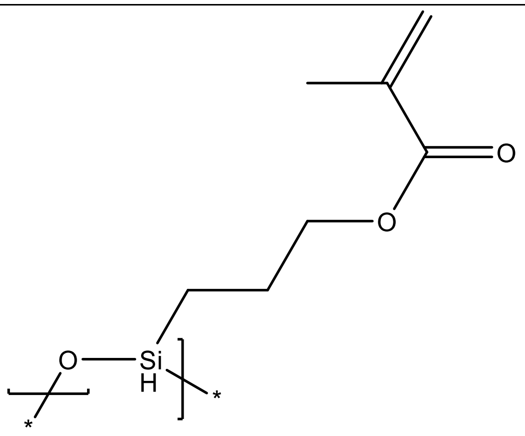
53	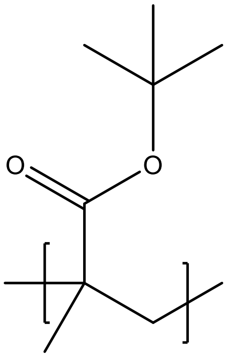
54	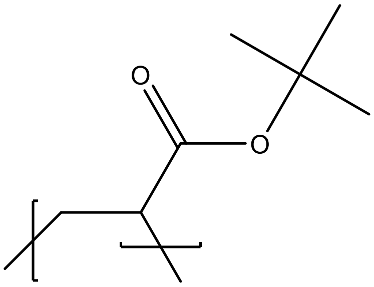
55	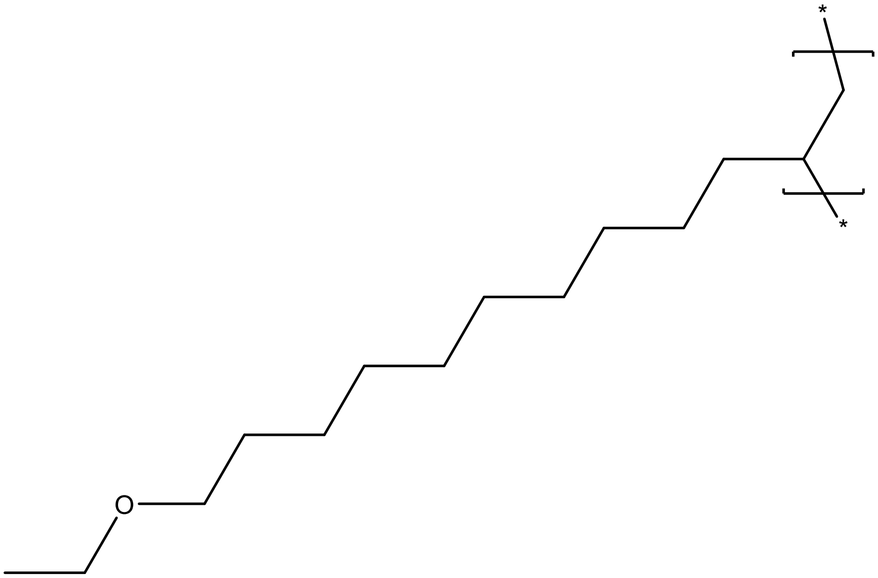

56	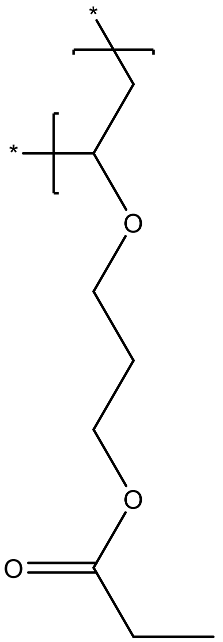
57	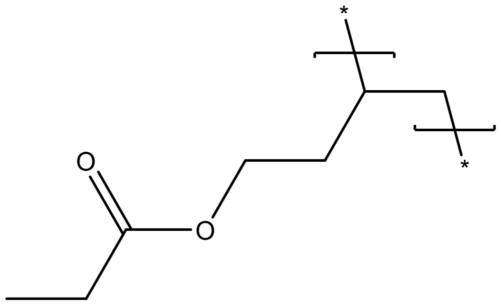
58	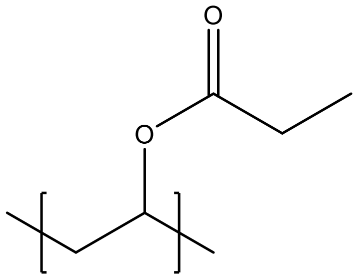
59	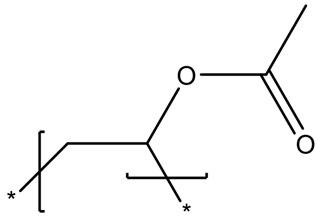
60	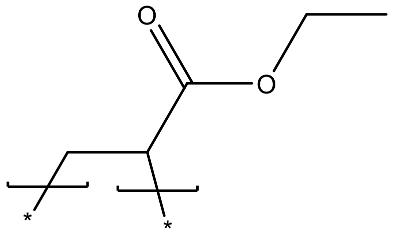

61	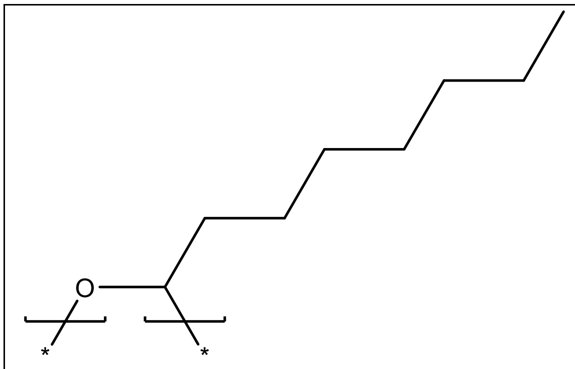
62	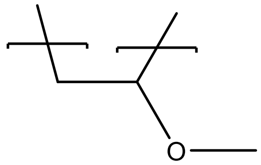
63	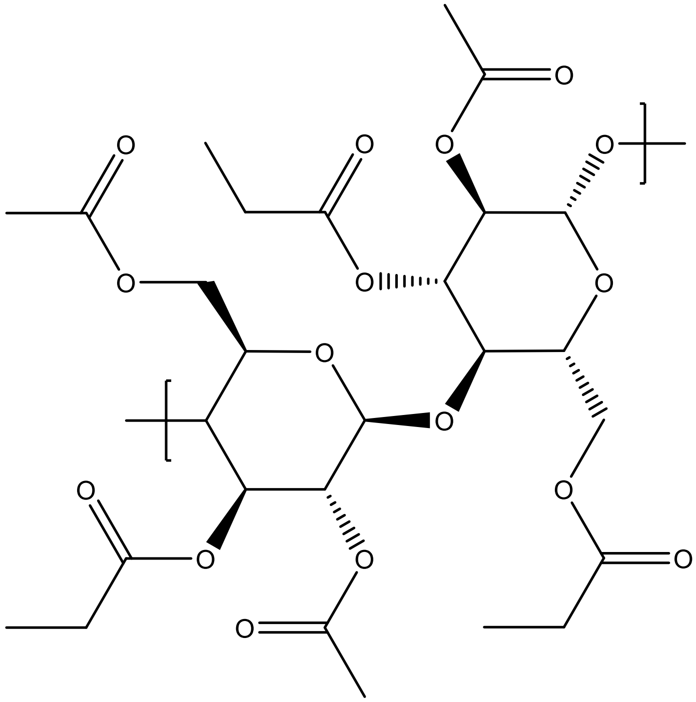
64	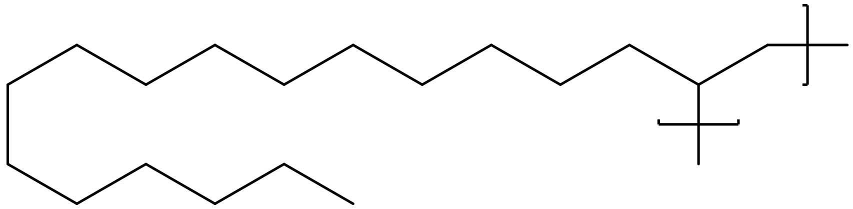

65

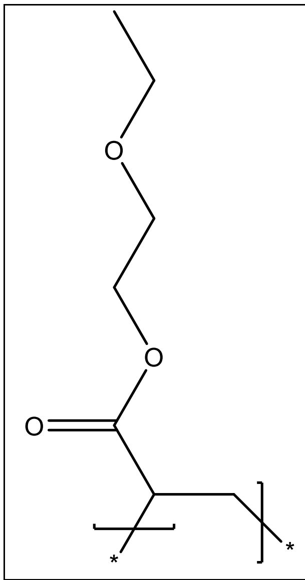

66

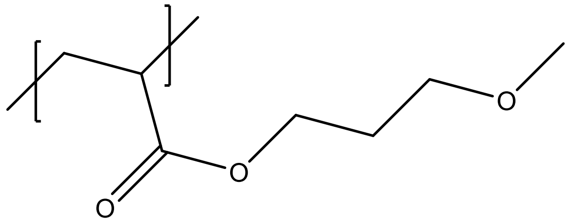

67

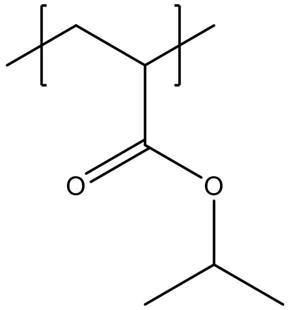

68

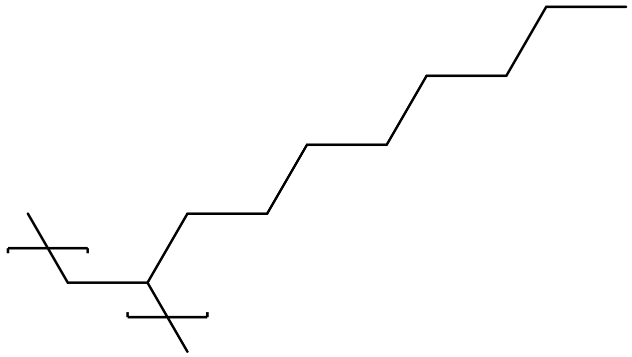

69

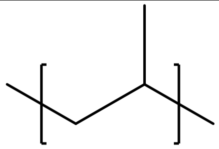

70	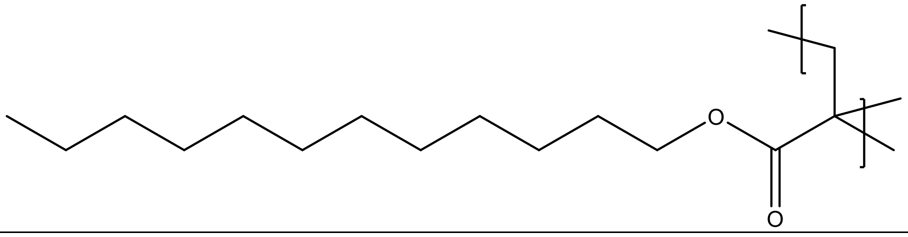
71	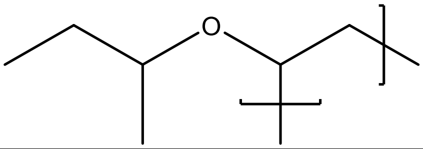
72	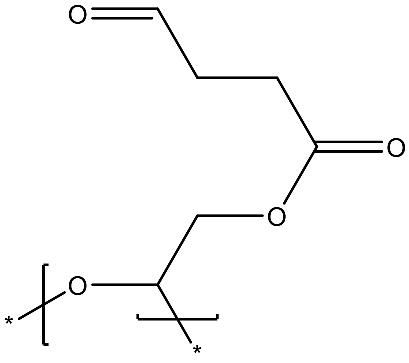
73	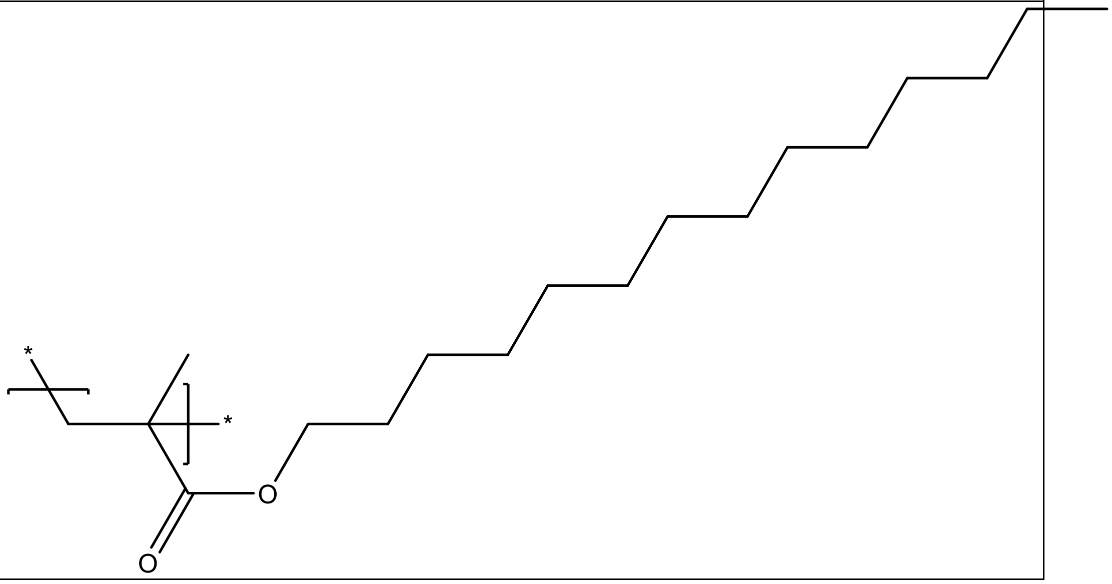

74	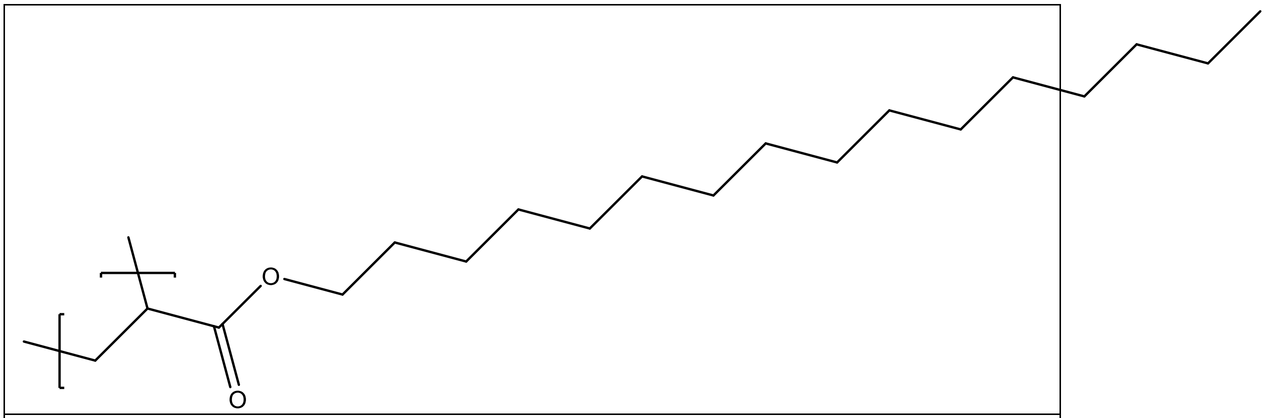
75	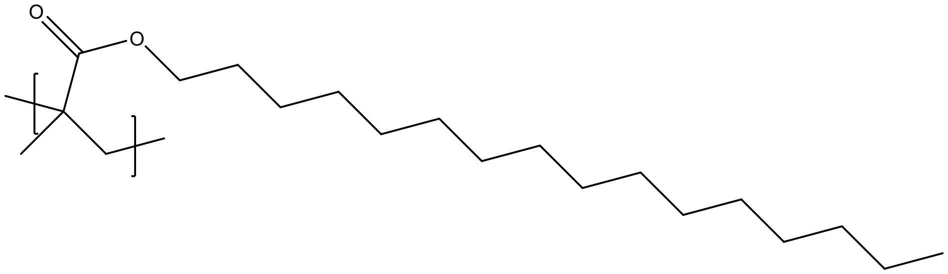
76	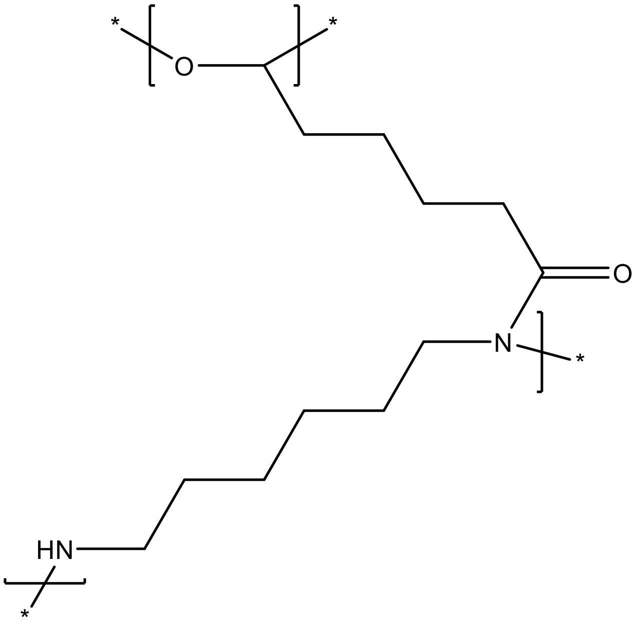

77	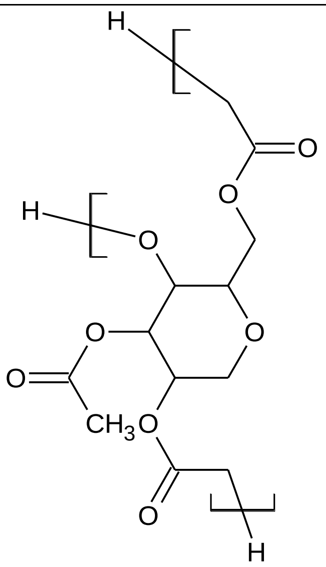
78	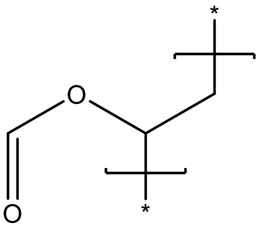
79	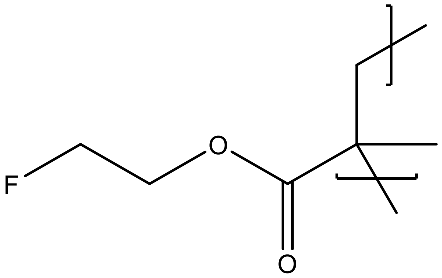
80	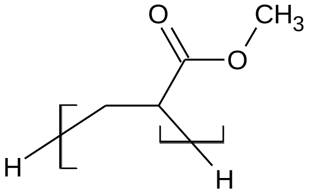

81
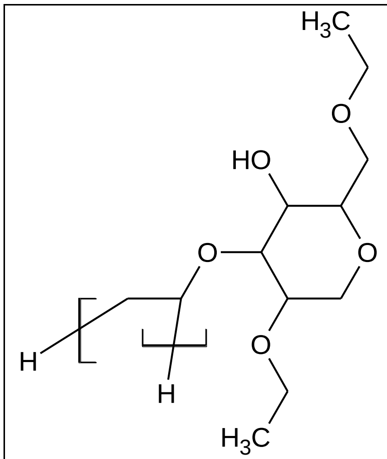

82
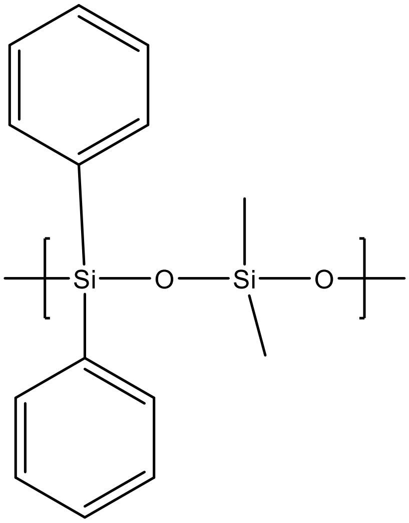

83
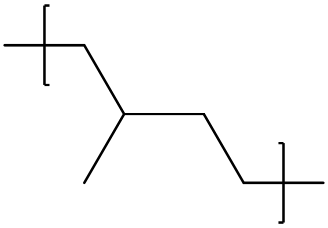

84
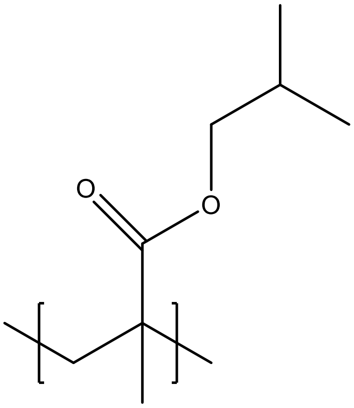

85	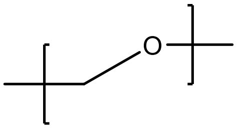
86	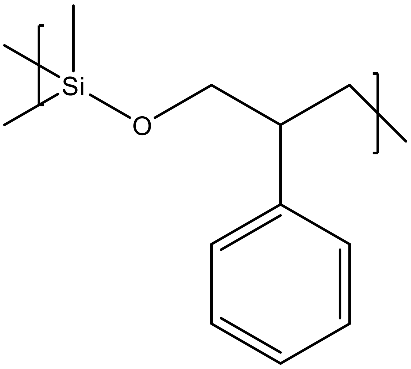
87	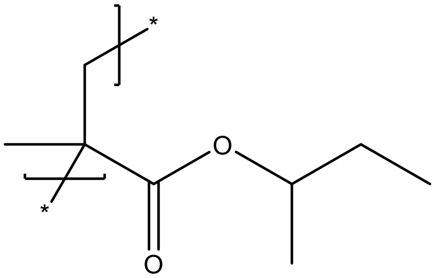
88	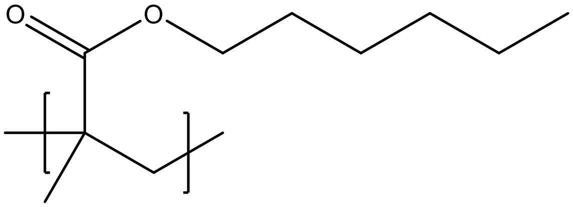
89	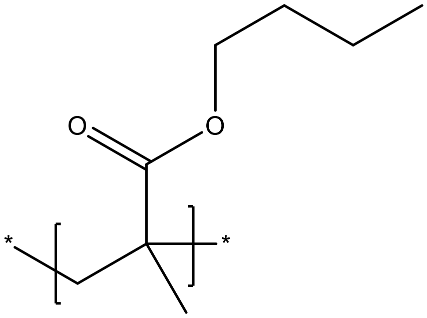

90	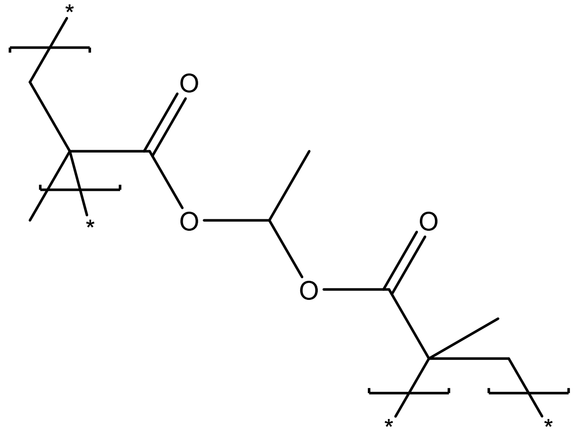
91	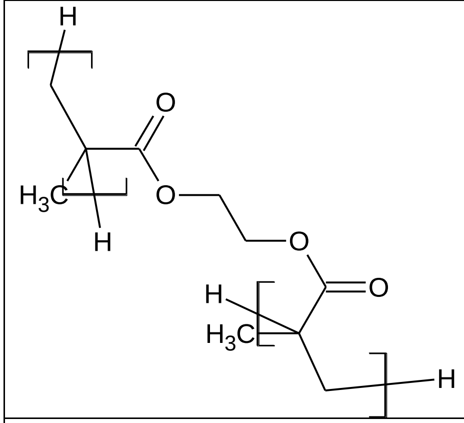
92	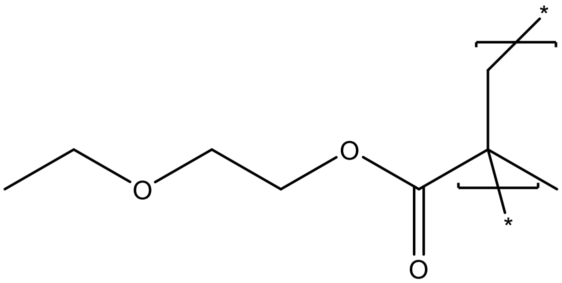
93	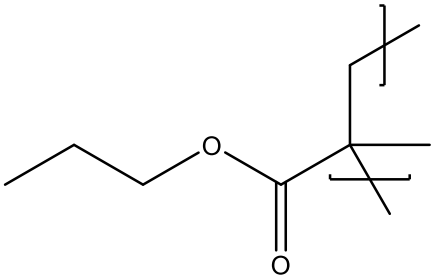
94	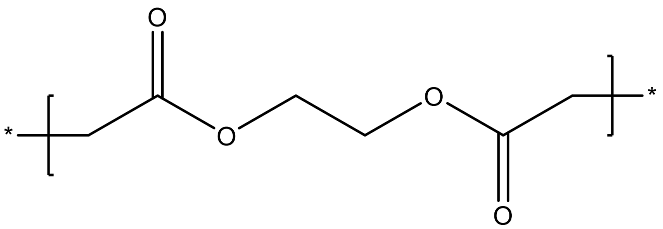

95

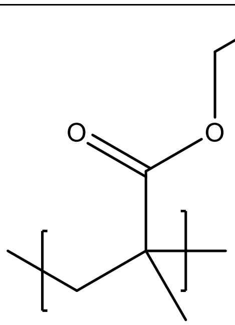

96

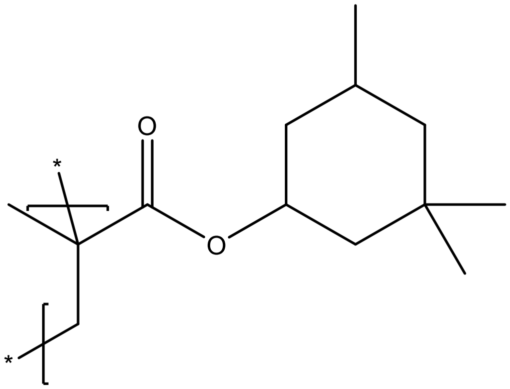

97

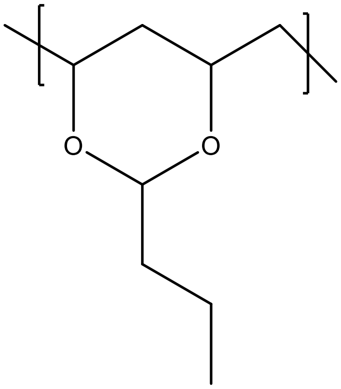

98

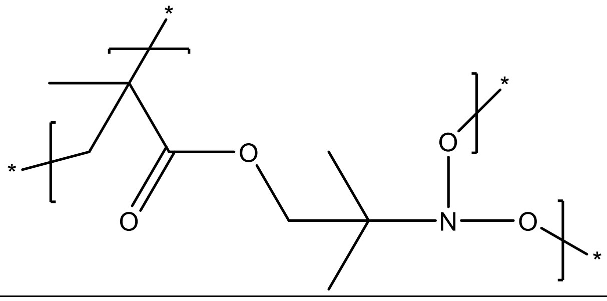

99

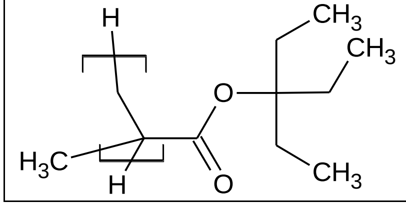

100	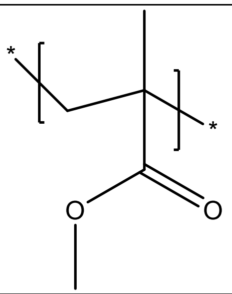
101	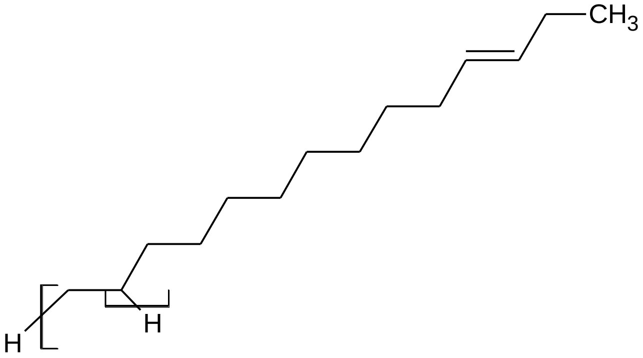
102	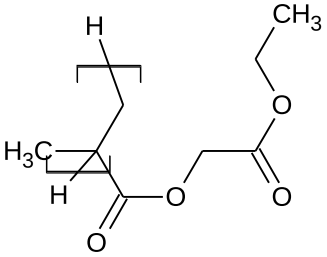
103	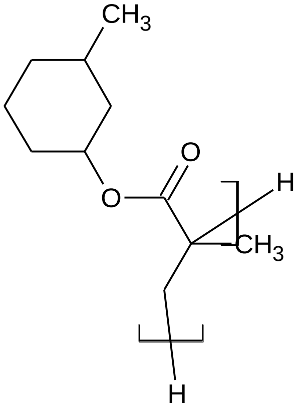
104	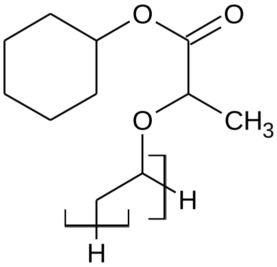

105	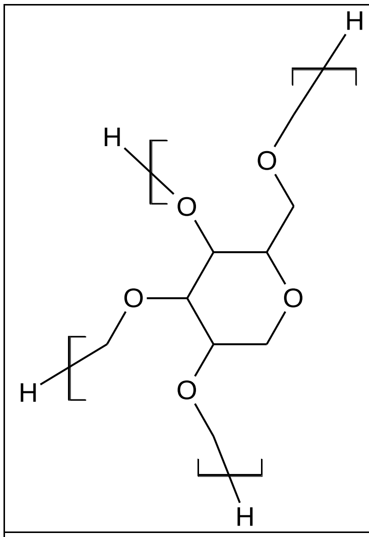
106	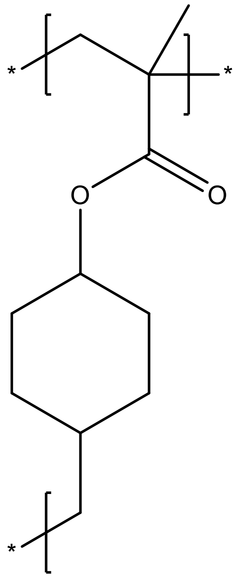
107	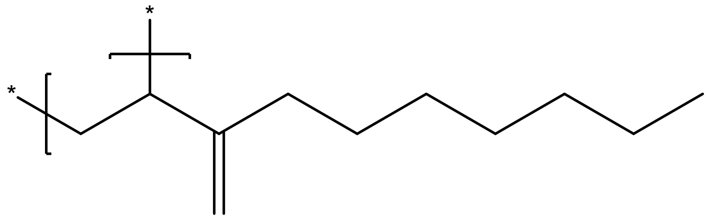
108	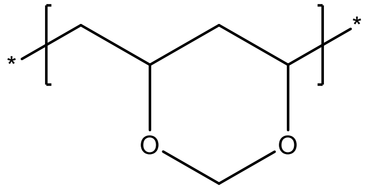

109	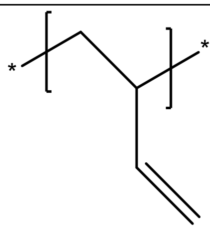
110	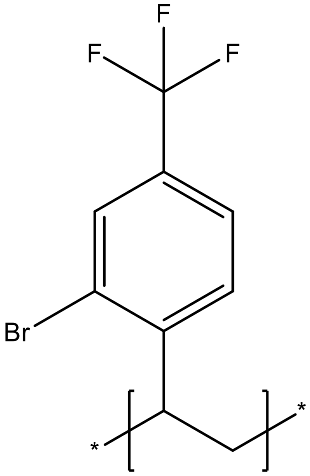
111	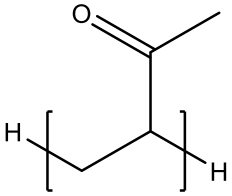
112	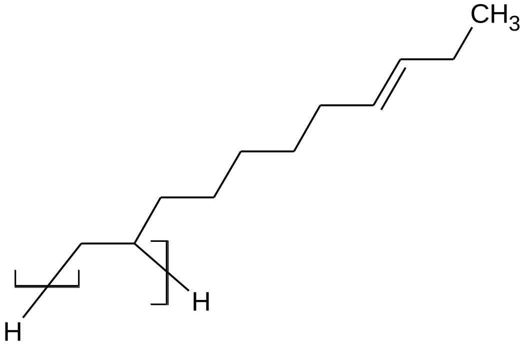
113	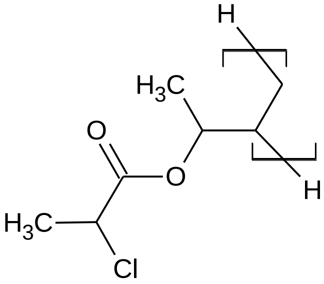

114	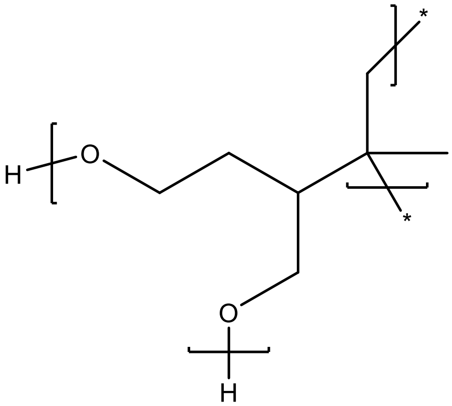
115	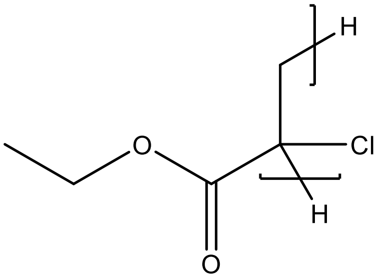
116	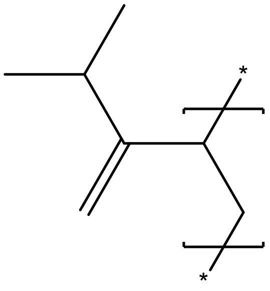
117	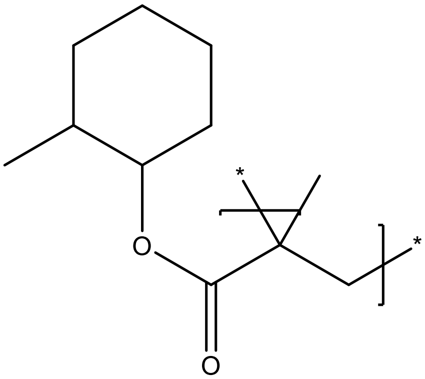
118	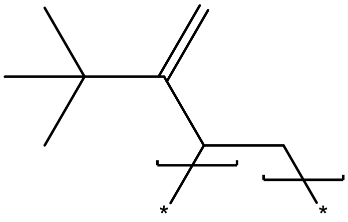

119	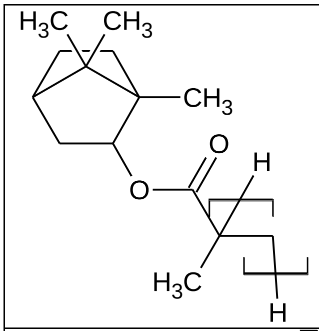
120	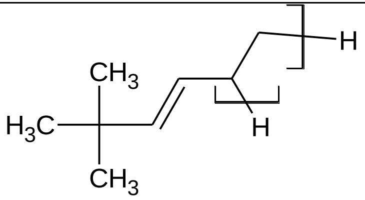
121	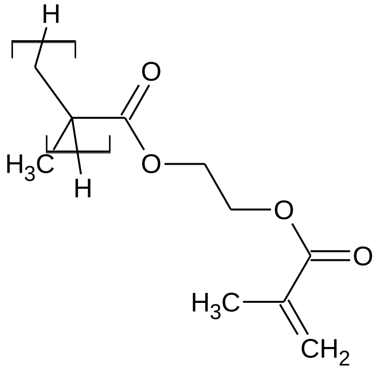
122	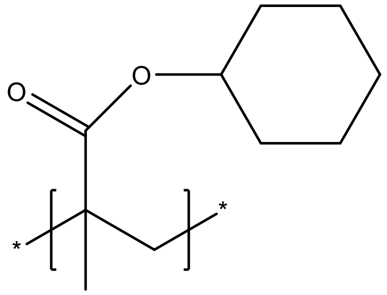

123	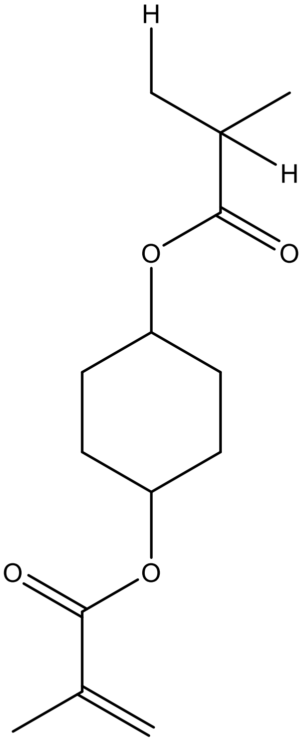
124	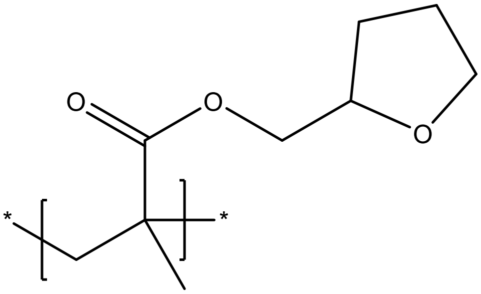
125	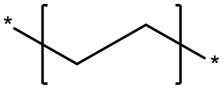
126	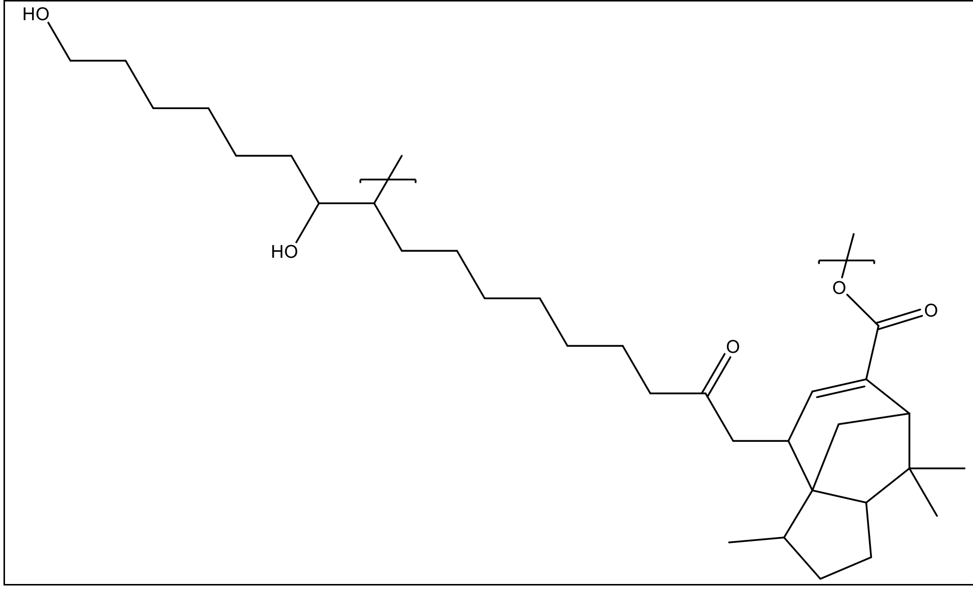

127	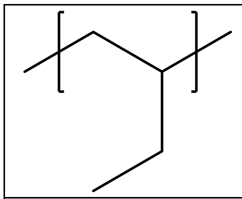
128	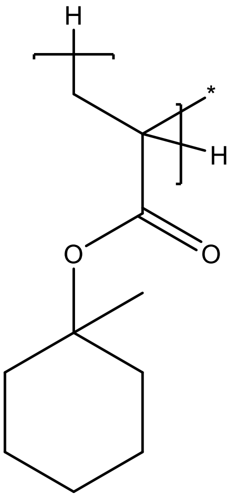
129	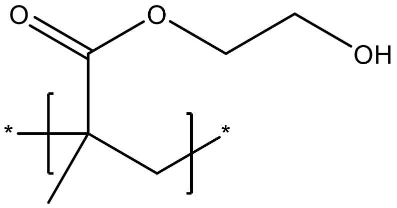
130	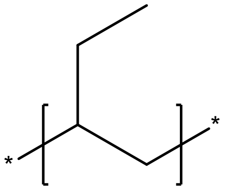
131	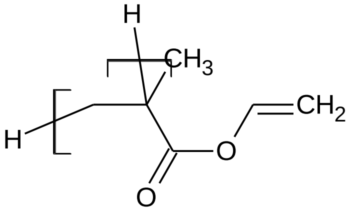
132	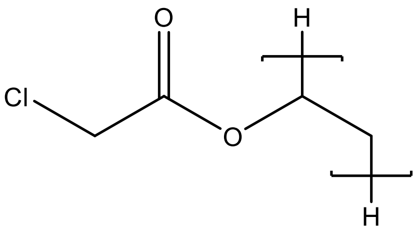
133	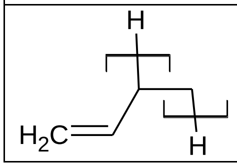

134	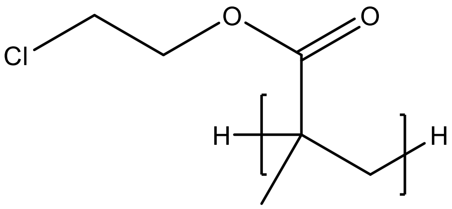
135	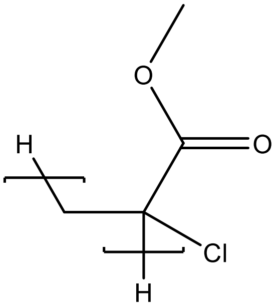
136	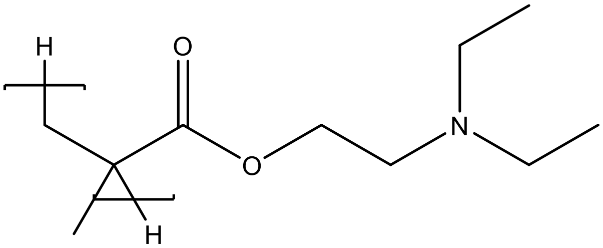
137	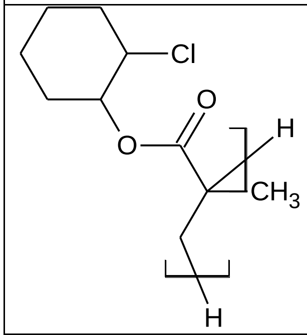
138	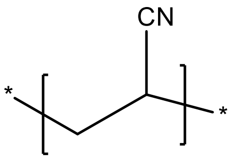
139	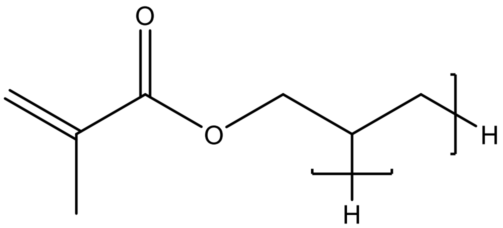
140	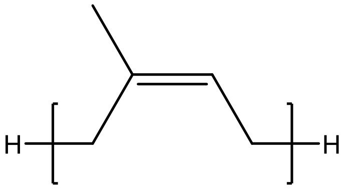

141
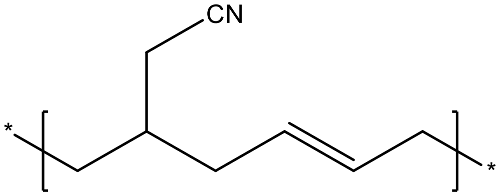

142
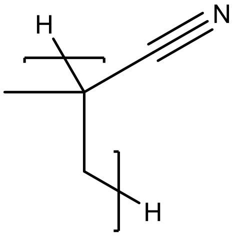

143
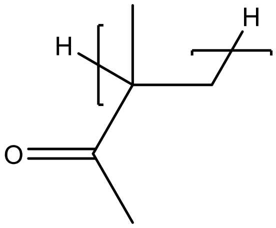

144
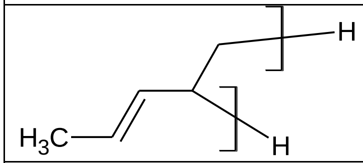

145
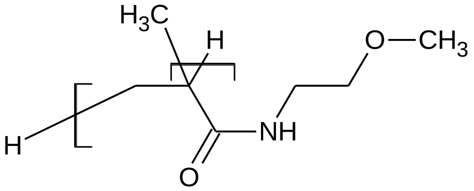

146
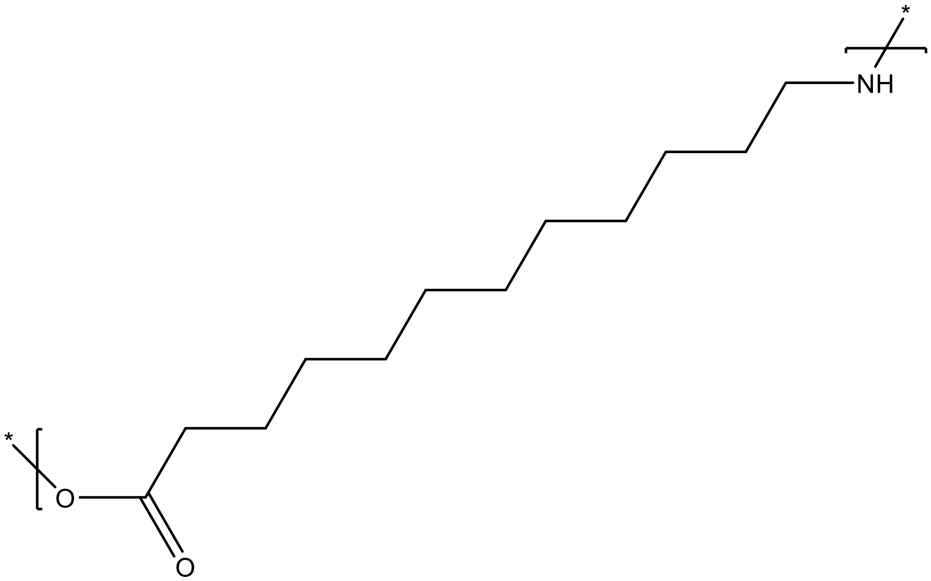

147	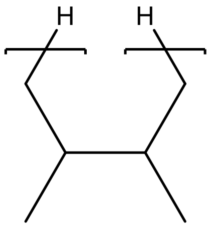
148	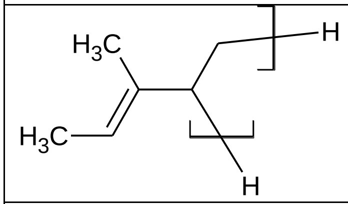
149	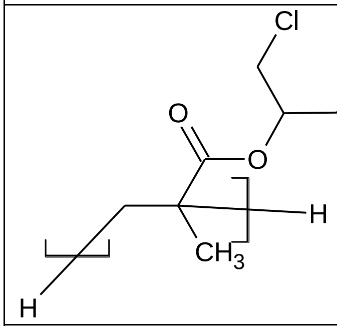
150	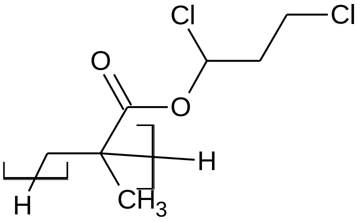
151	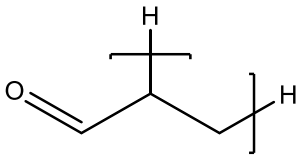
152	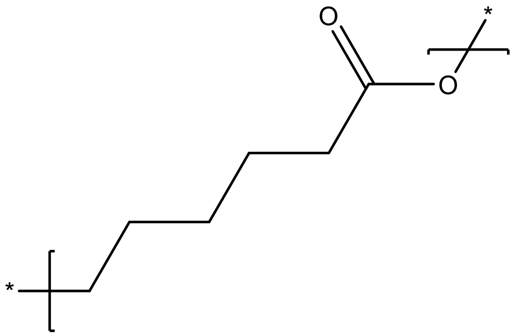
153	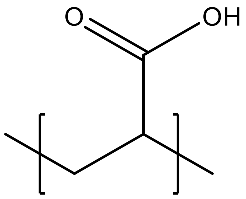

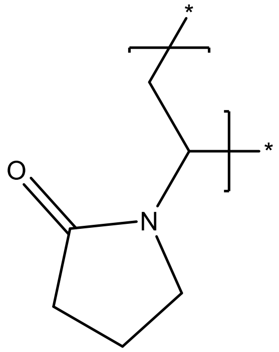

154

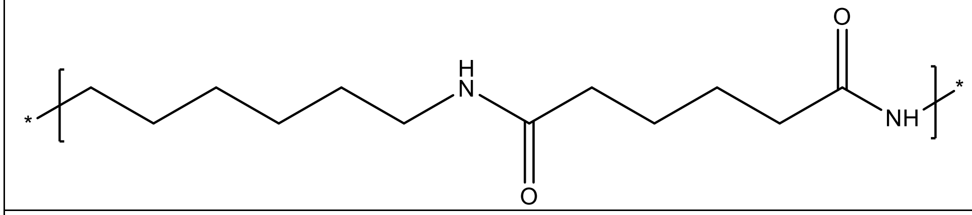

155

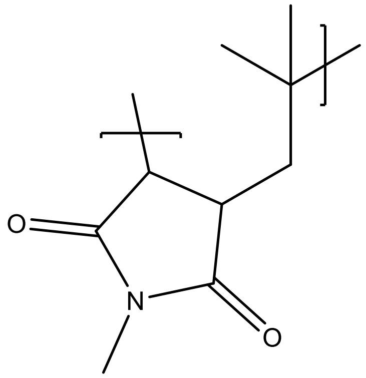

156

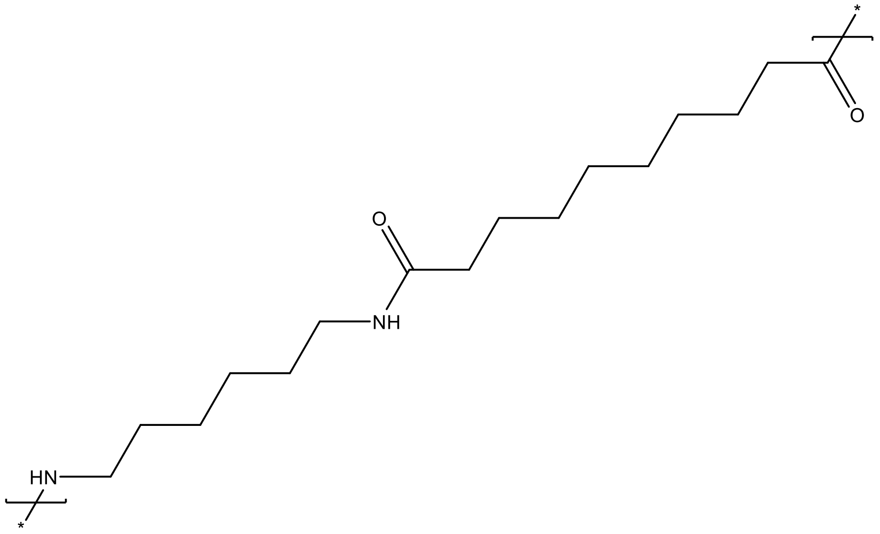

157

158	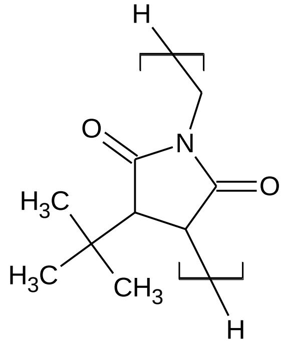
159	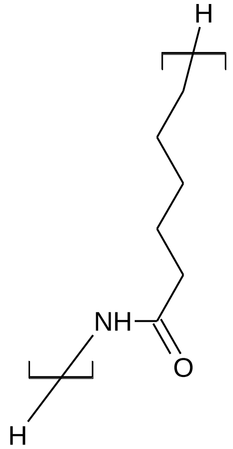
160	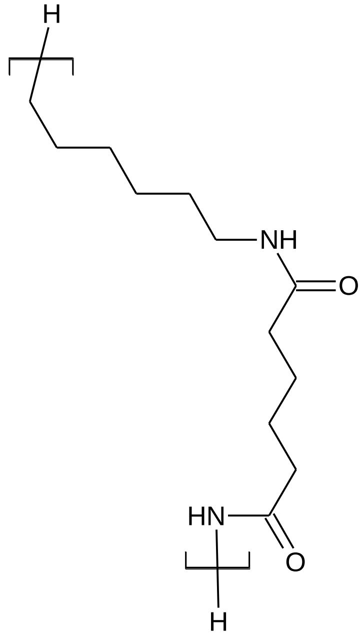

161	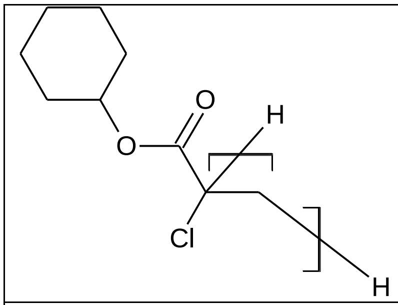
162	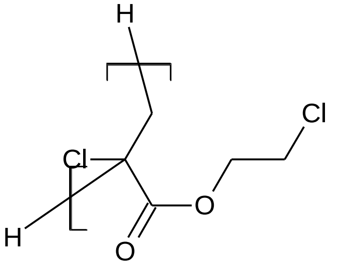
163	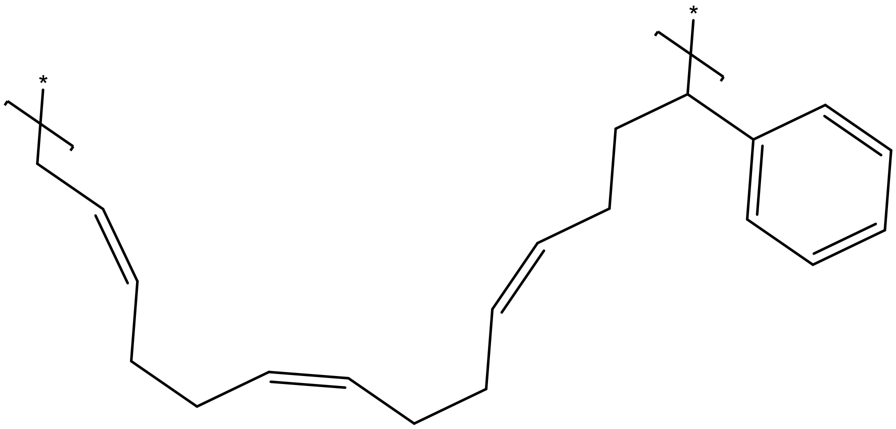
164	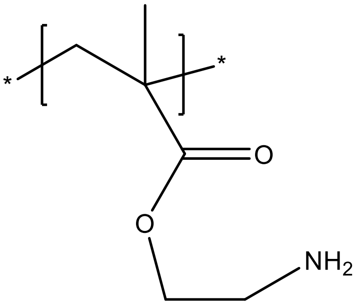
165	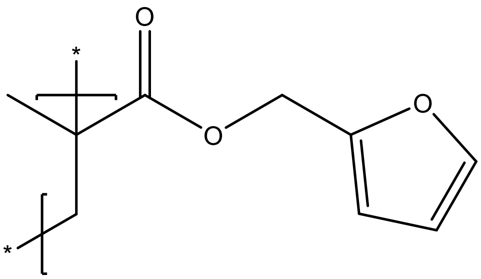

166

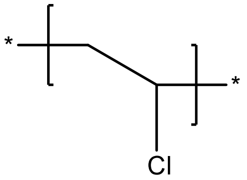

167

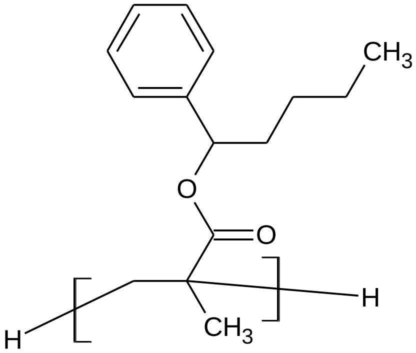

168

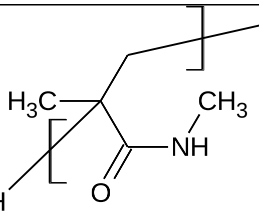

169

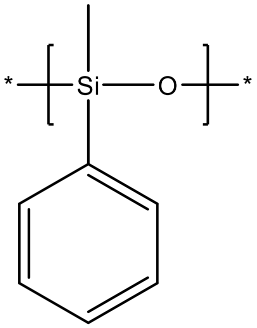

170

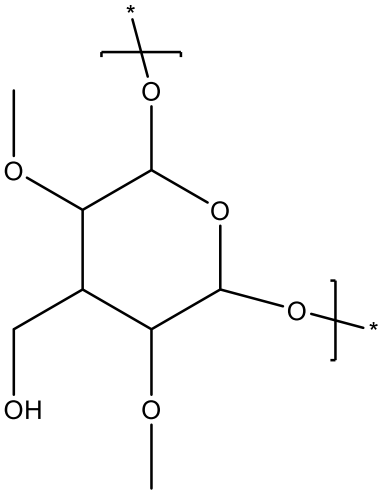

171	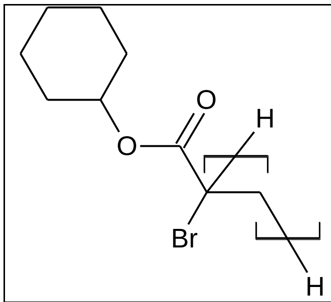
172	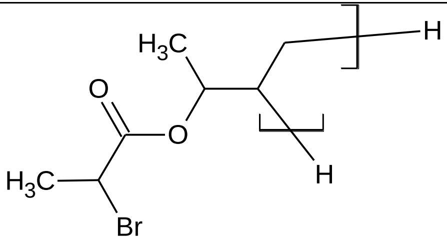
173	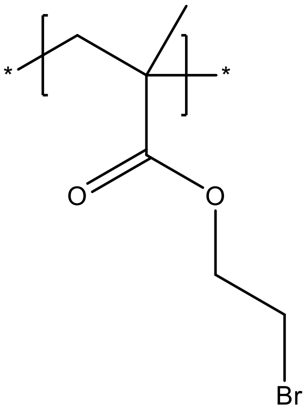
174	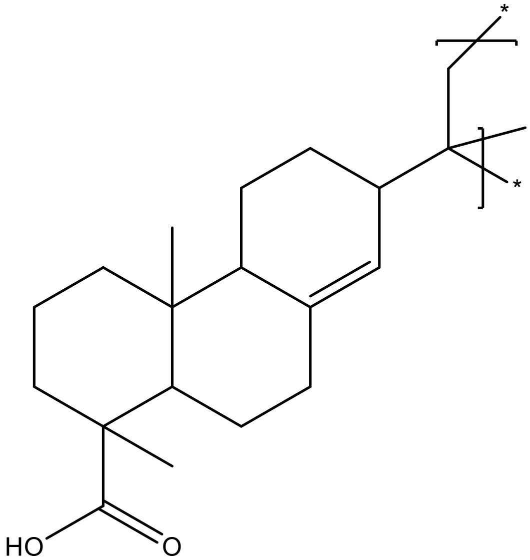

175	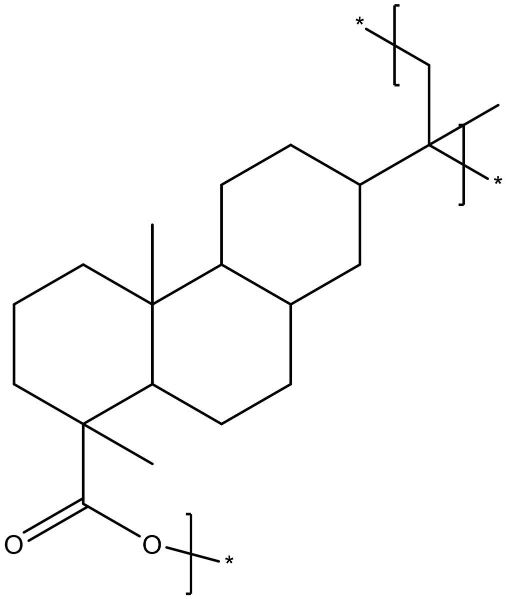
176	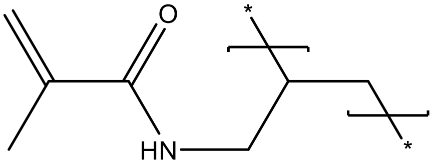
177	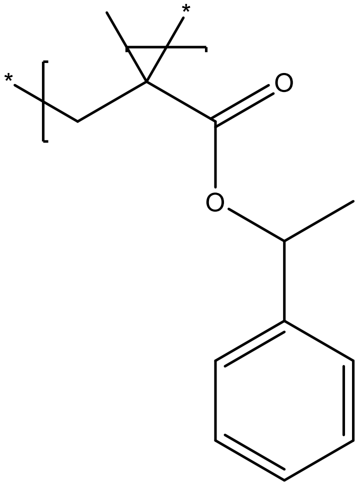
178	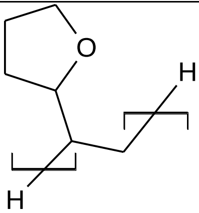
179	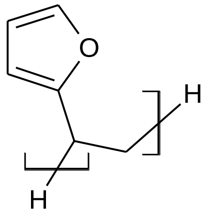

180	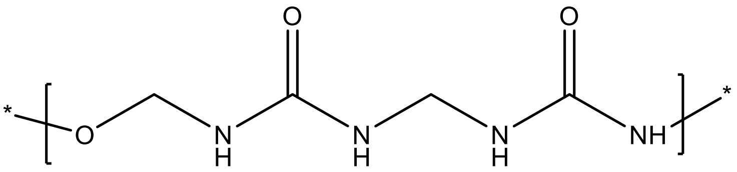
181	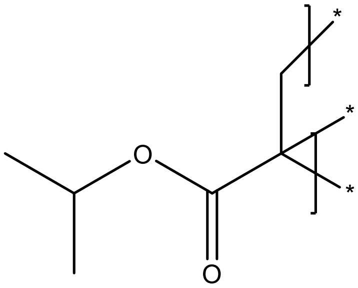
182	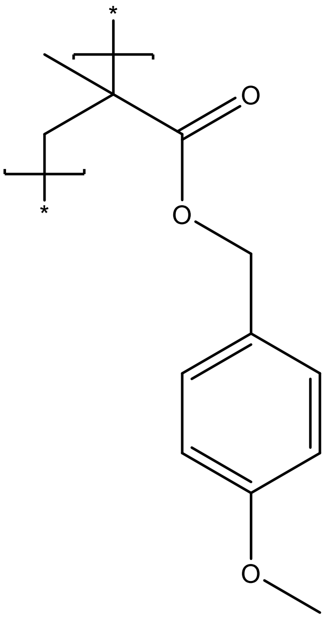
183	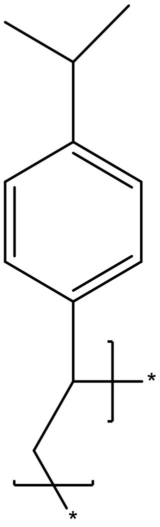

184	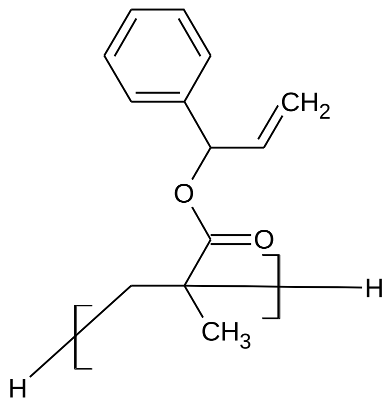
185	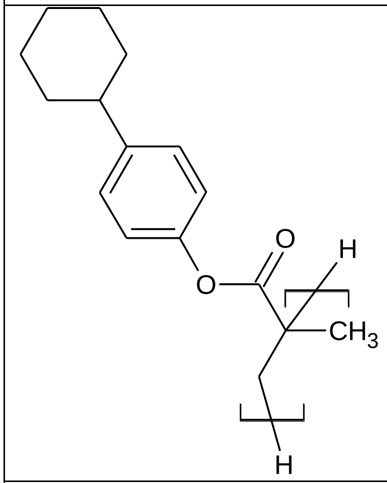
186	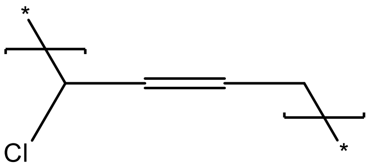
187	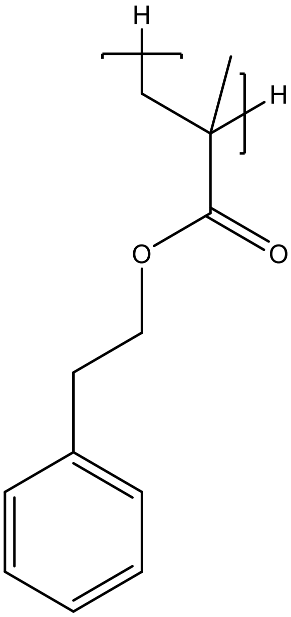

188	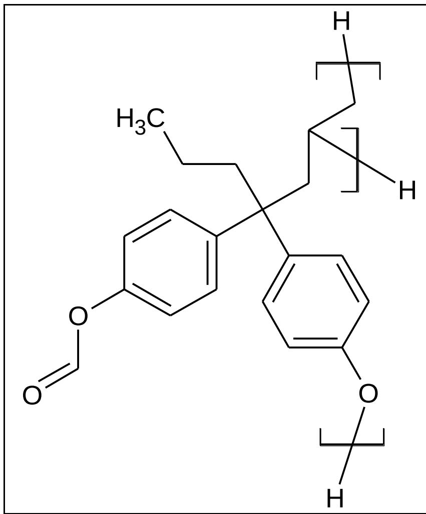
189	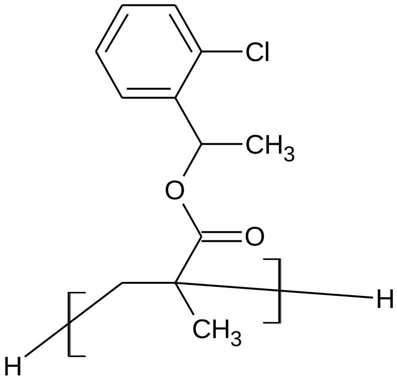
190	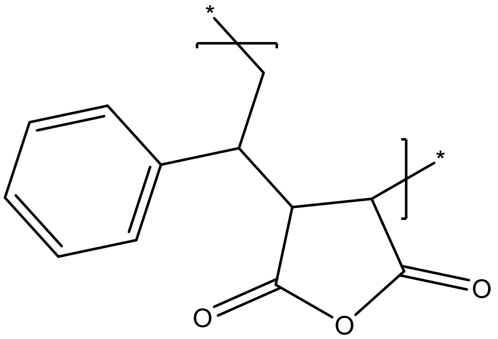
191	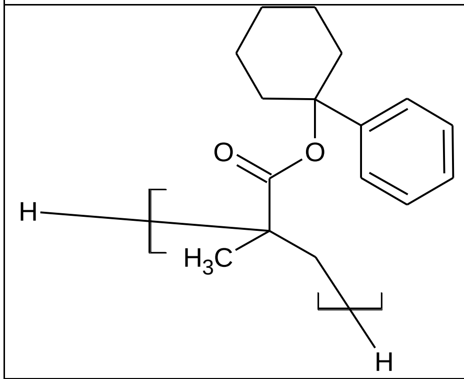

192	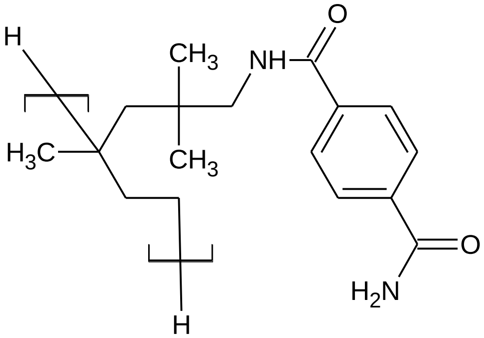
193	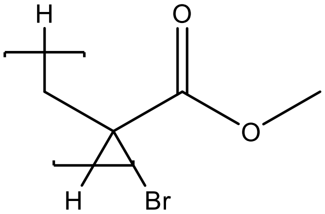
194	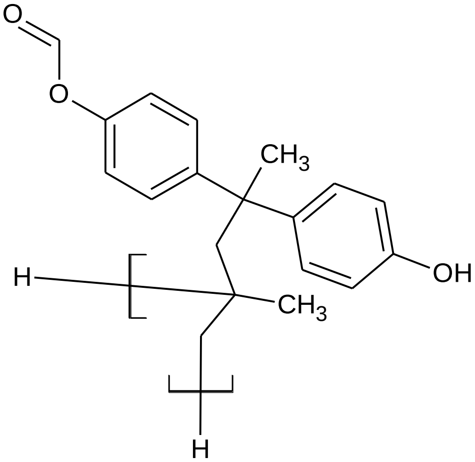
195	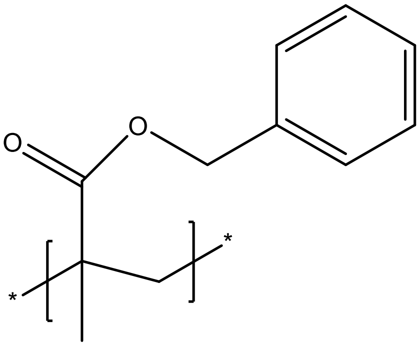

196	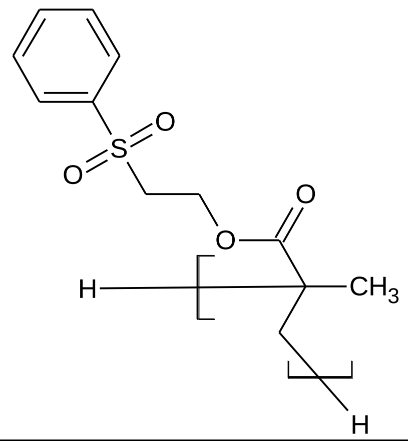
197	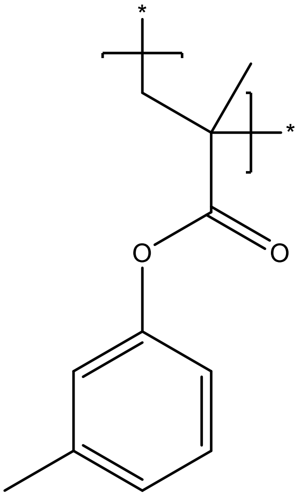
198	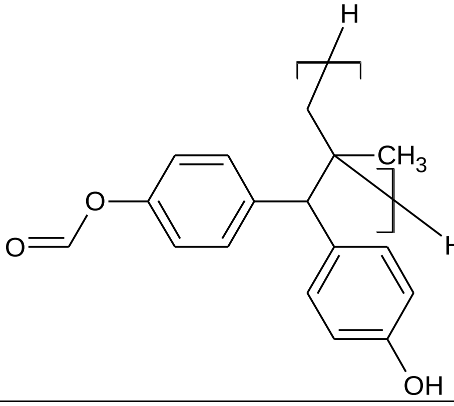
199	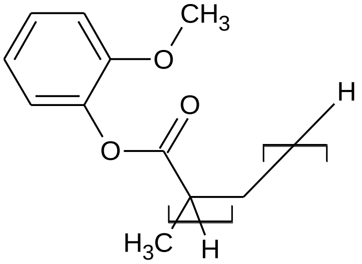

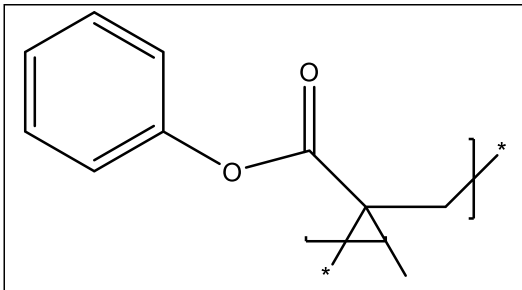

200

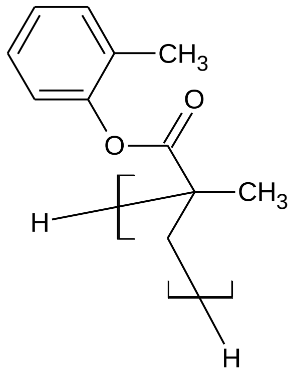

201

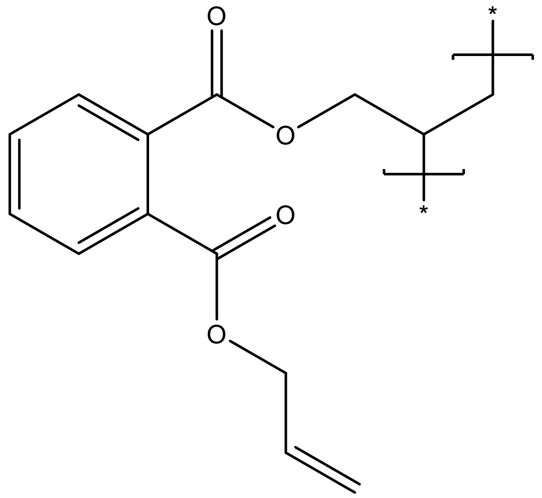

202

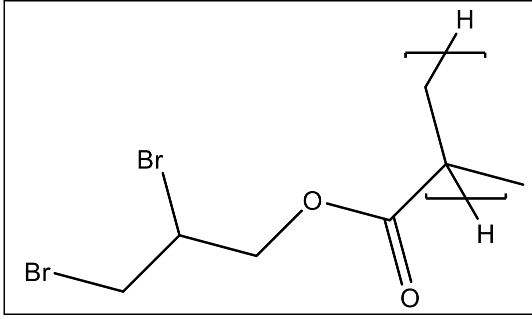

203

204
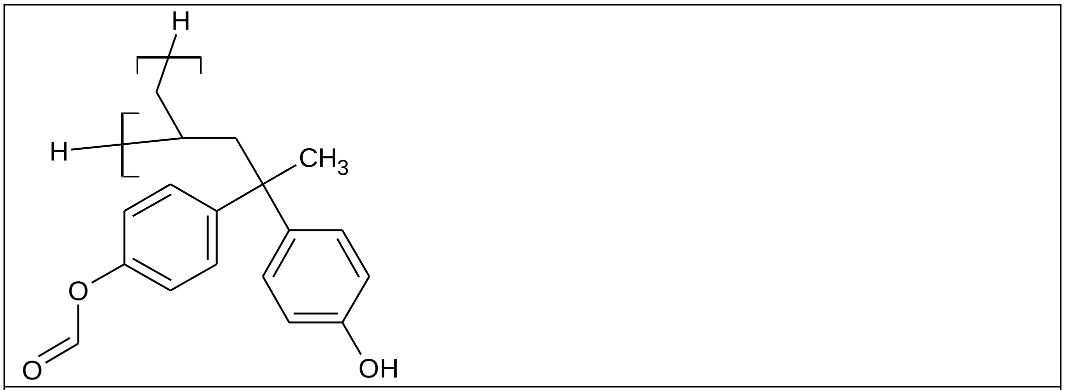

205
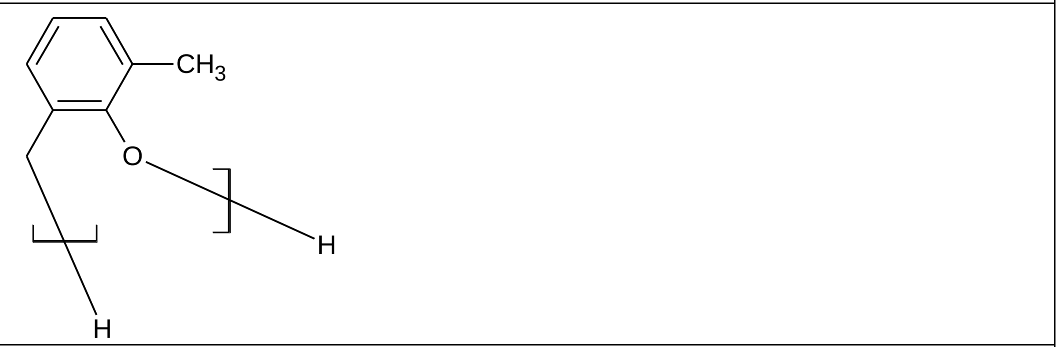

206
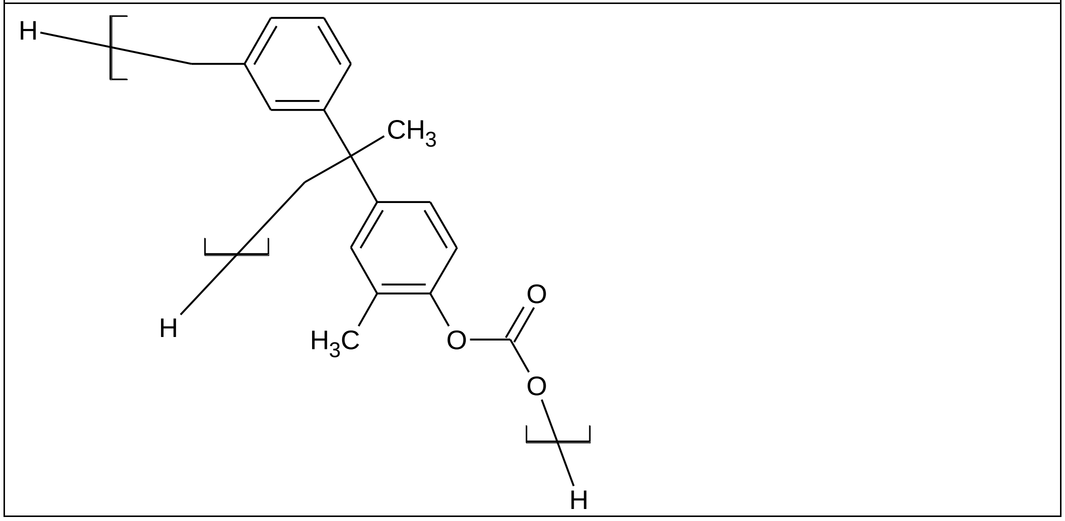

207	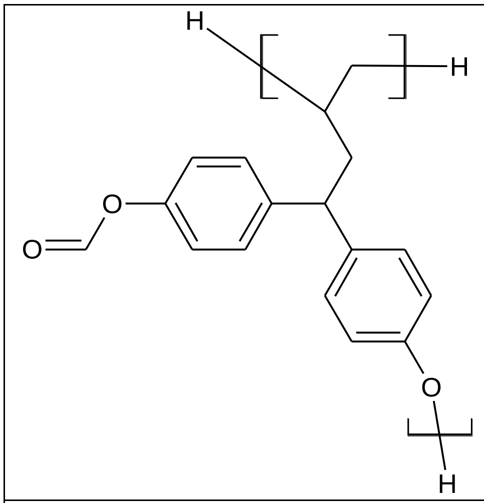
208	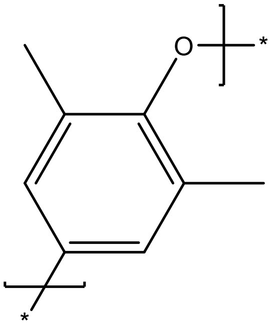
209	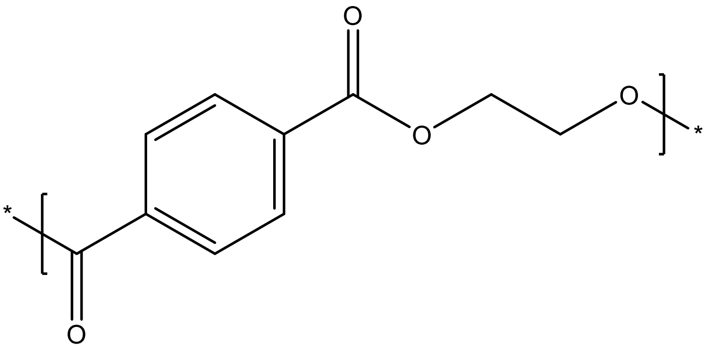
210	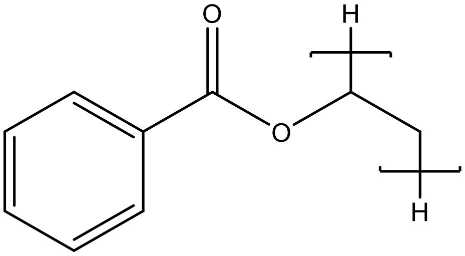
211	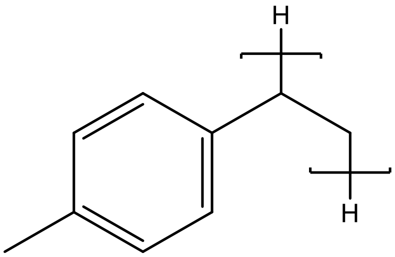

212	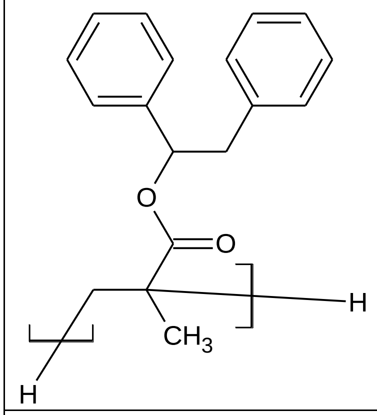
213	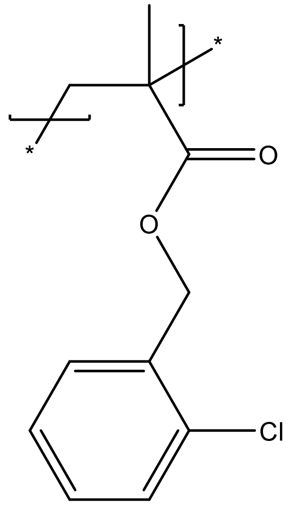
214	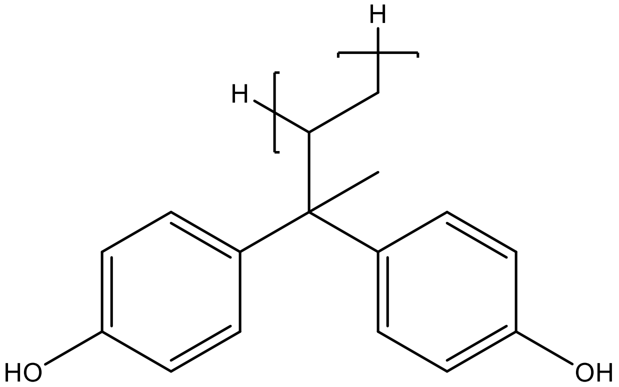

215	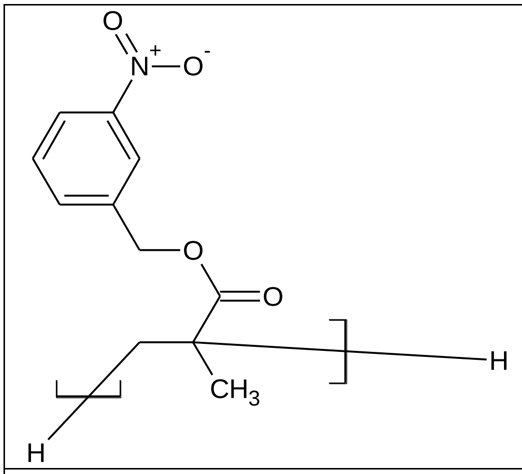
216	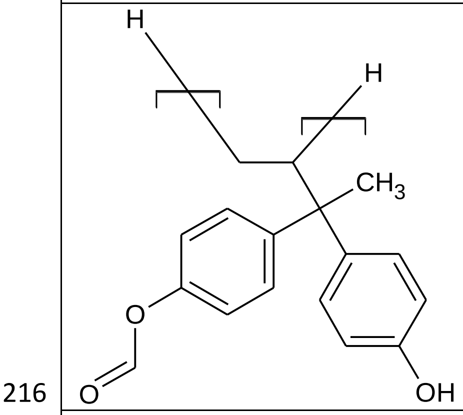
217	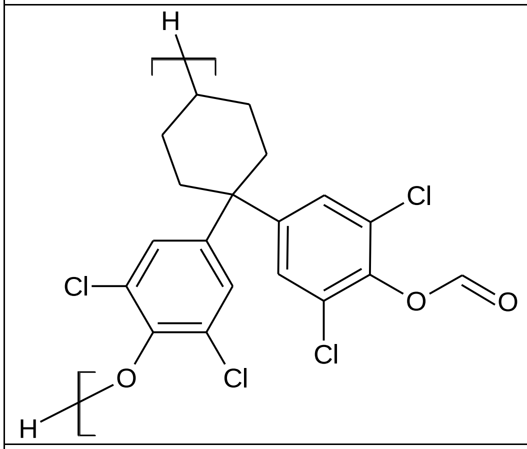
218	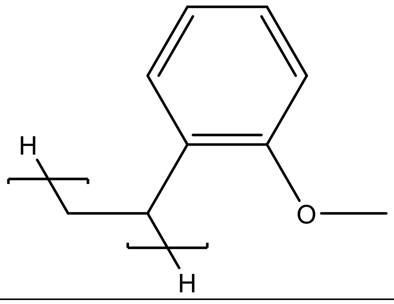

219	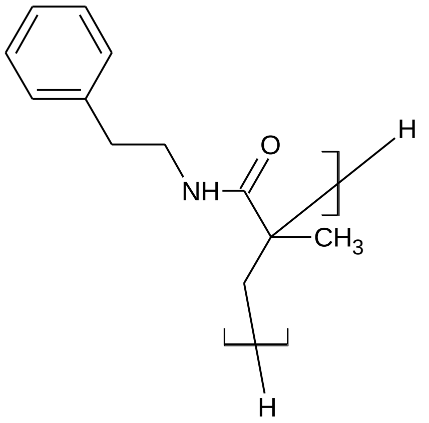
220	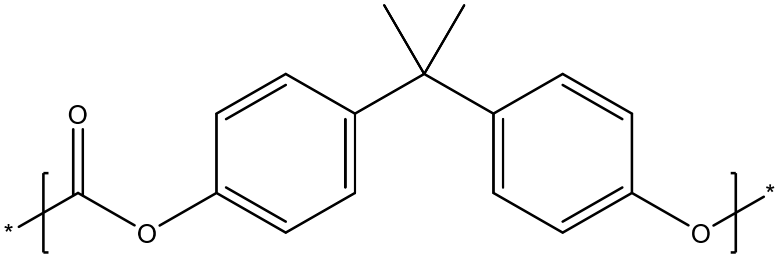
221	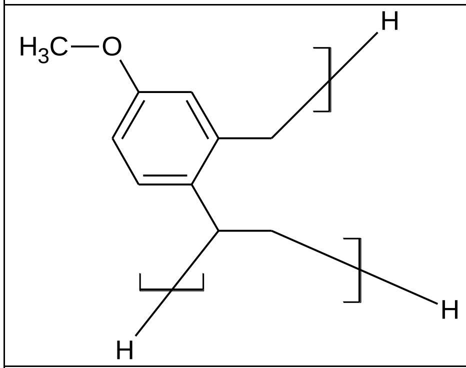
222	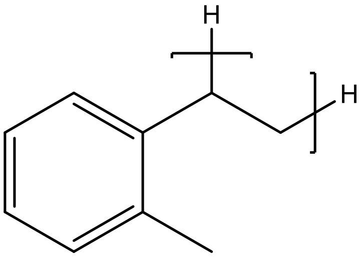
223	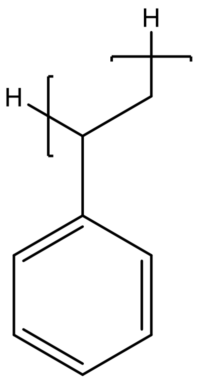

224	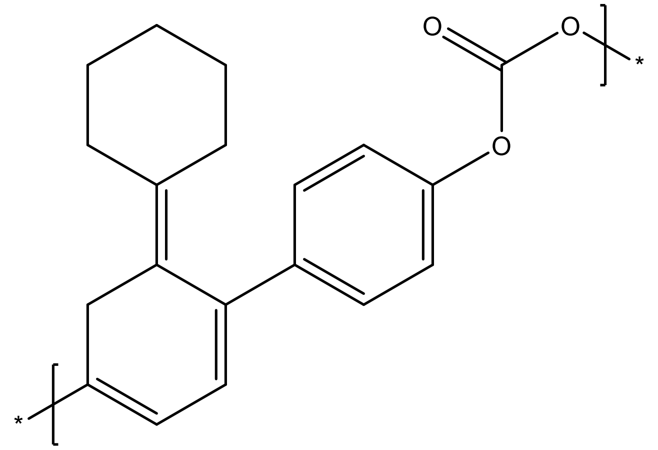
225	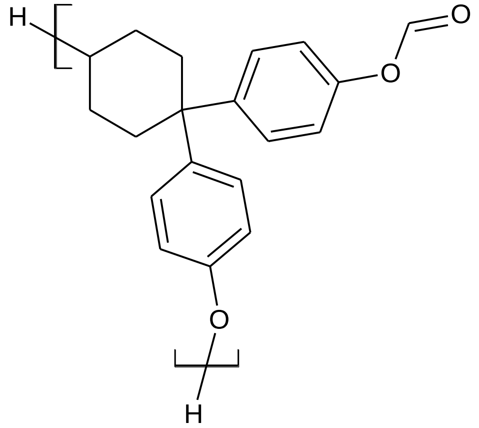
226	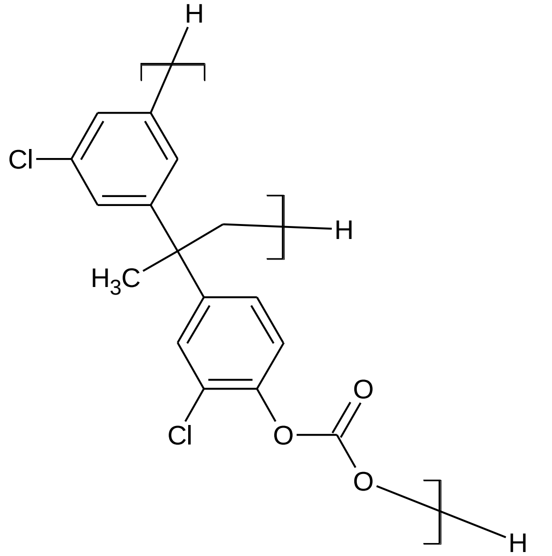

227	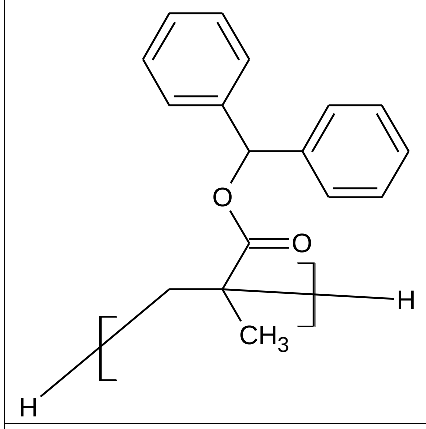
228	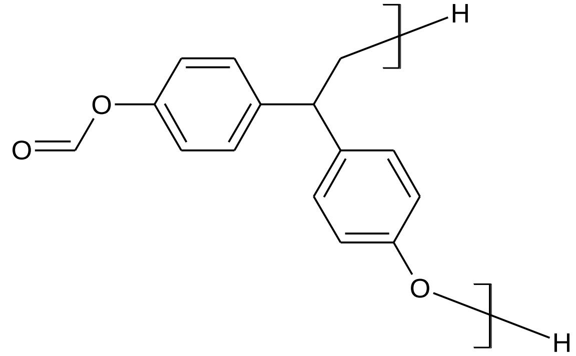
229	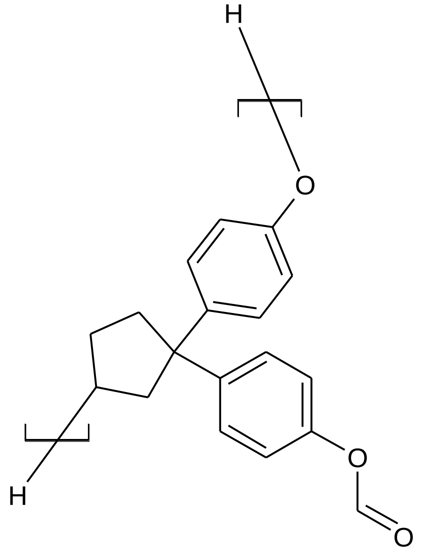

230	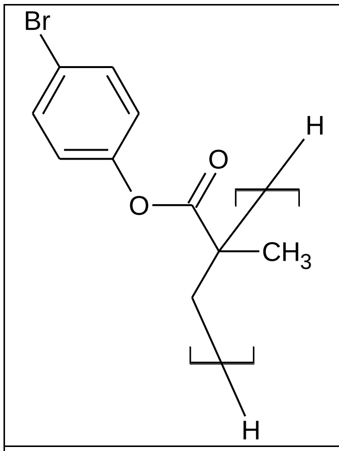
231	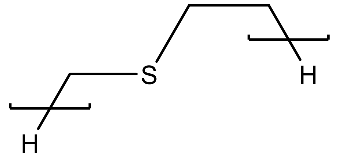
232	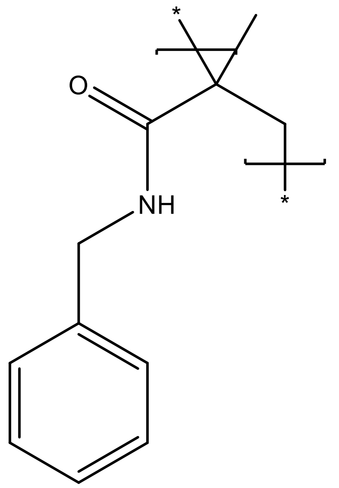
233	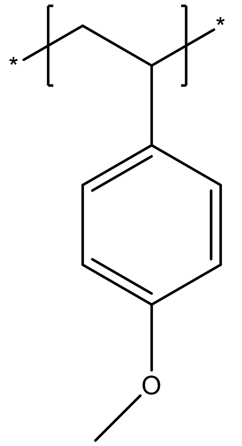

234	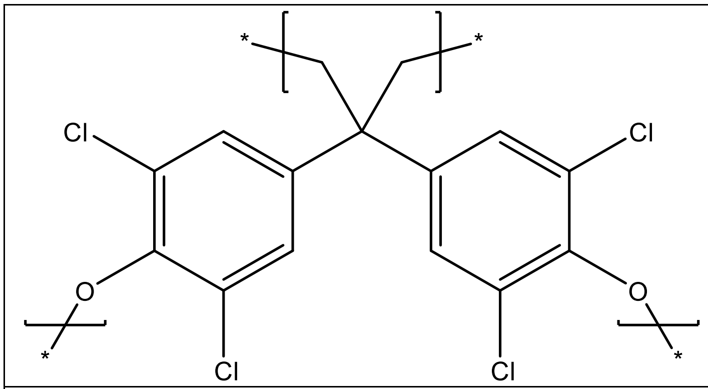
235	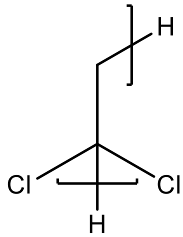
236	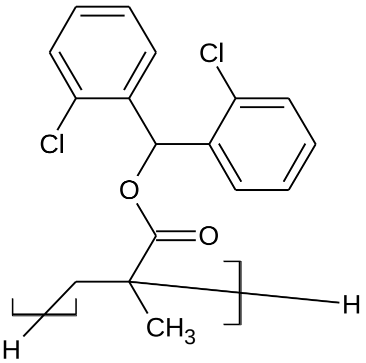
237	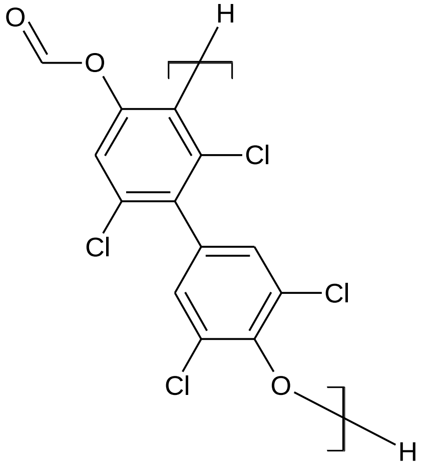

238	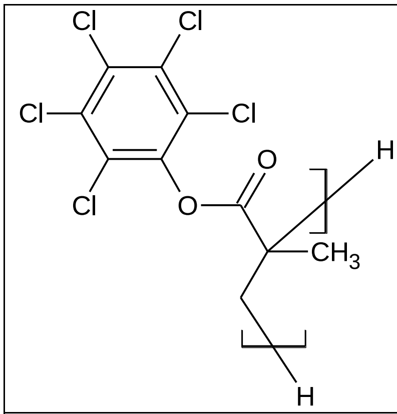
239	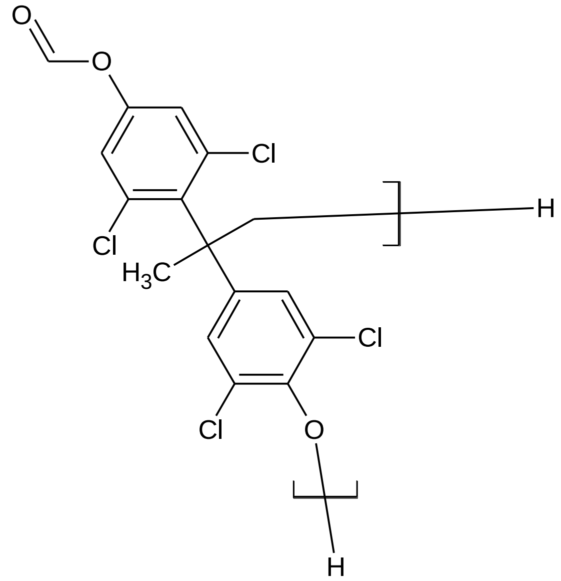
240	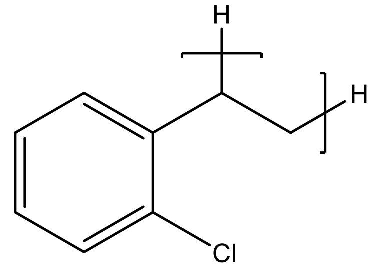
241	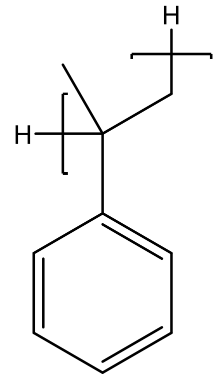

242	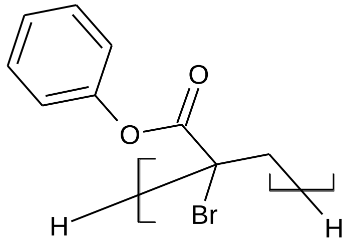
243	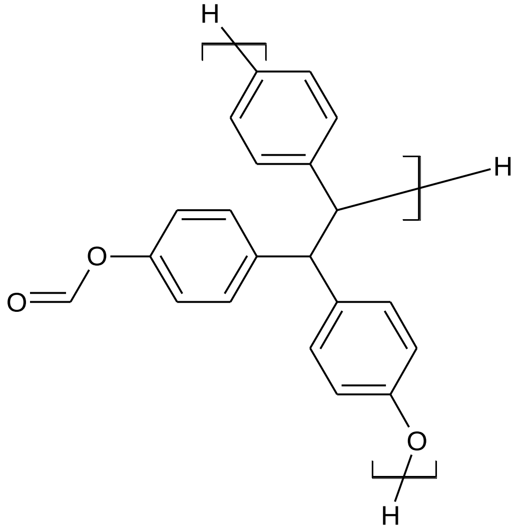
244	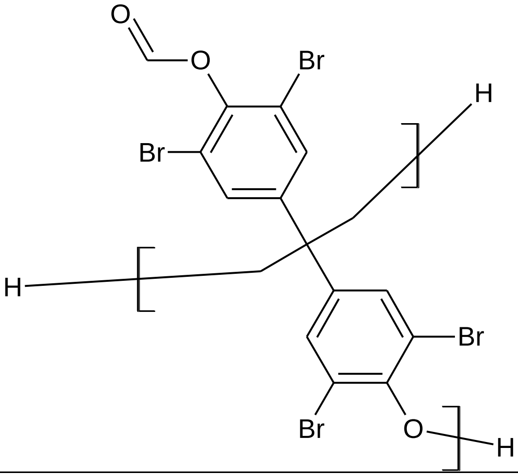
245	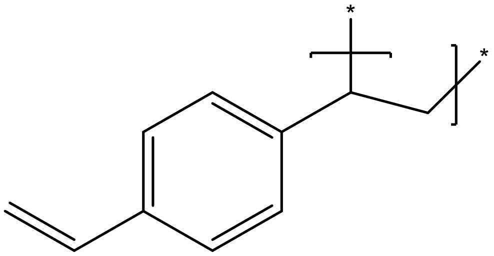

246	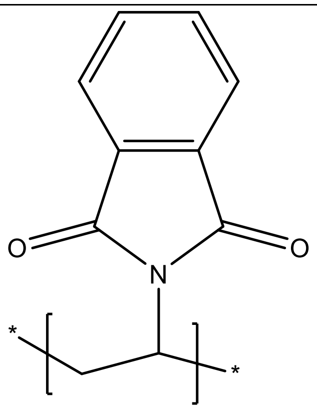
247	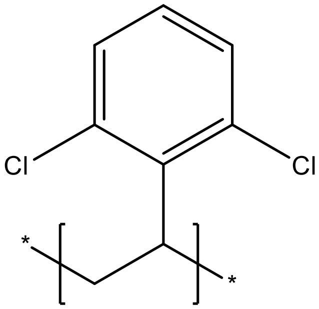
248	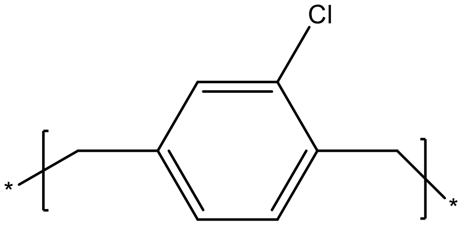
249	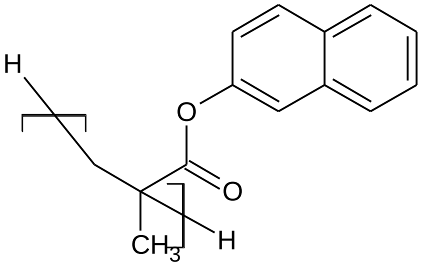
250	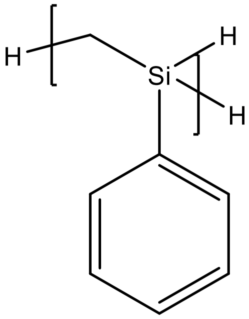

251	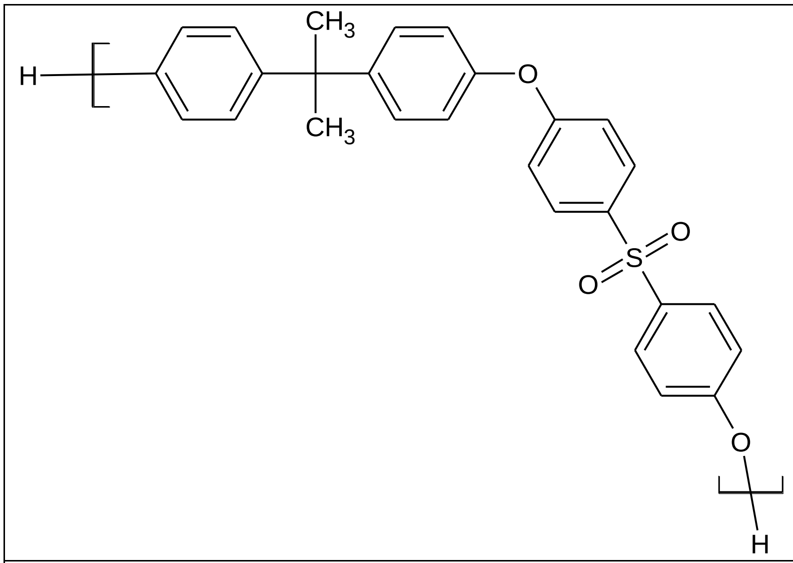
252	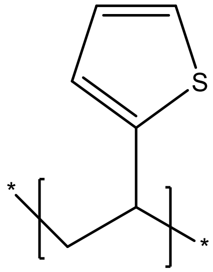
253	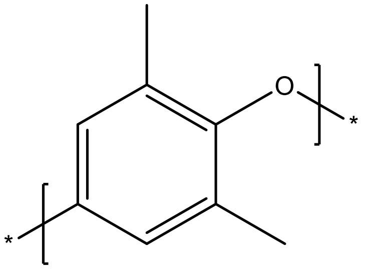
254	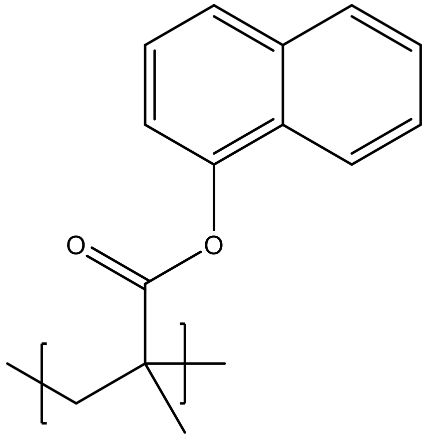

255	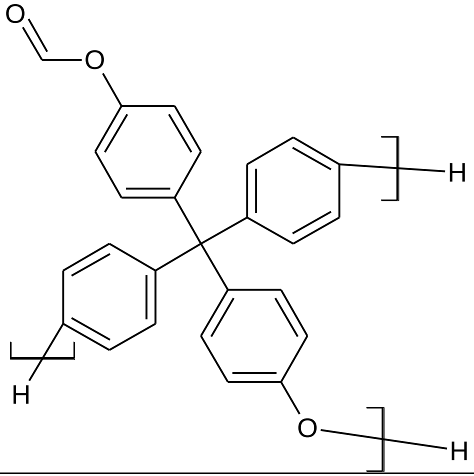
256	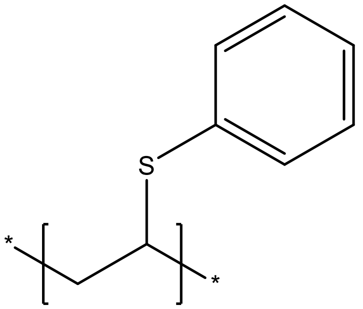
257	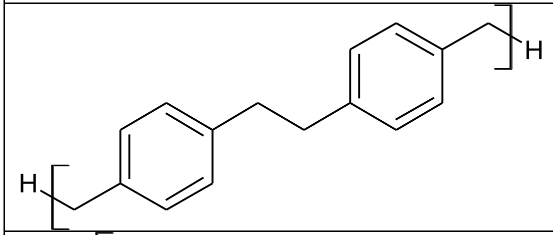
258	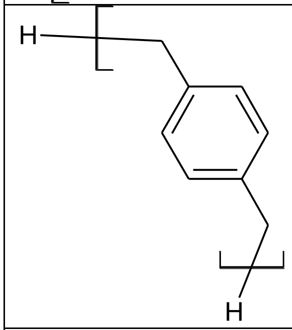
259	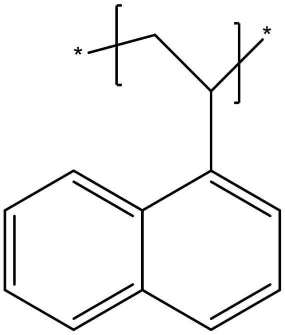

260	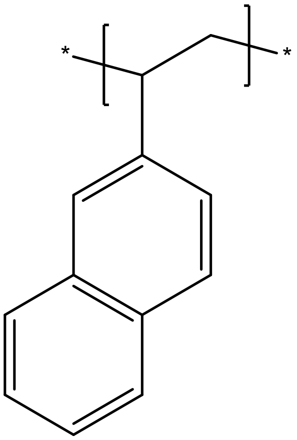
261	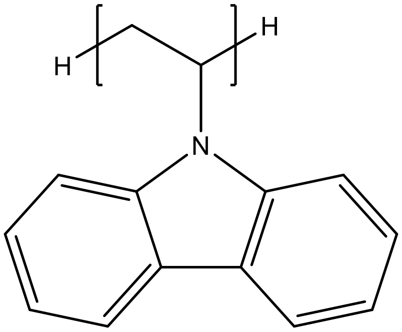
262	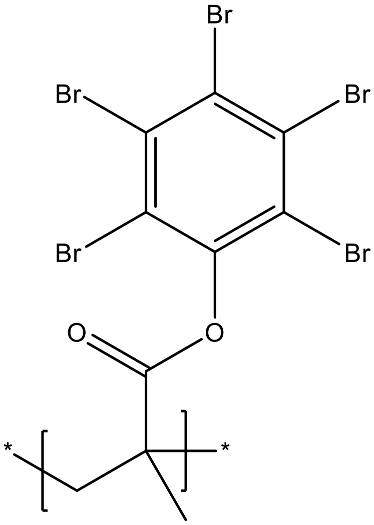

Table S2 - Number, polymer name, Smiles notation of end-capped monomer, the experimental refractive indices before and after log transformation, predicted log transformation from 4 variable model, and which set each molecule was a part of.

#	**Polymer Name**<br><br>***Smiles END-CAPPED***	**Exp. RI**	**Exp. Log RI**	**Pred. Log RI**	**Set**
1	Poly(hexafluoropropylene oxide)<br><br>*OC(F)(F)[C@H](F)C(F)(F)F*	1.31	0.117	0.115	Training
2	Poly(tetrafluoroethylene-co-hexafluoropropylene)<br><br>*C(C(C(C(F)C(F)(F)F)(F)F)(F)F)(F)F*	1.34	0.126	0.112	Training
3	Poly (pentadecafluorooctyl acrylate)<br><br>*C(CC)(O)OCC(C(C(C(C(C(C(CF)(F)F)(F)F)(F)F)(F)F)(F)F)(F)F)(F)F*	1.34	0.127	0.130	Training
4	Poly(tetrafluoro-3-(heptafluoropropoxy)propyl acrylate)<br><br>*C(=O)(C[CH2])OC(C(COC(C(C(F)(F)F)(F)F)(F)F)(F)F)(F)F*	1.35	0.129	0.127	Training
5	Poly(tetrafluoro-3-(pentafluoroethoxy)propyl acrylate)<br><br>*C(=O)(CC)OC(C(COC(C(F)(F)F)(F)F)(F)F)(F)F*	1.35	0.130	0.129	Prediction
6	Poly(tetrafluoroethylene)<br><br>*C(C(F)F)(F)F*	1.35	0.130	0.119	Training
7	Poly(undecafluorohexyl acrylate)<br><br>*C(=O)(CC)OC(C(C(C(C(CF)(F)F)(F)F)(F)F)(F)F)(F)F*	1.36	0.132	0.131	Training
8	Poly(nonafluoropentyl acrylate)<br><br>*C(=O)(CC)OC(C(C(C(CF)(F)F)(F)F)(F)F)(F)F*	1.36	0.134	0.134	Training
9	Poly(tetrafluoro-3-(trifIuoromethoxy)propyl acrylate)<br><br>*CCC(=O)OCC(F)(C(F)(F)OC(F)(F)F)F*	1.36	0.134	0.133	Prediction
10	Poly(pentafluorovinyl propionate)<br><br>*C(=O)(CC)OC(C(CF)(F)F)(F)F*	1.36	0.135	0.145	Training
11	Poly(heptafluorobutyl acrylate)<br><br>*C(=O)(CC)OC(C(C(CF)(F)F)(F)F)(F)F*	1.37	0.136	0.139	Training
12	Poly(trifluorovinyl acetate)<br><br>*C(C(OC(=O)C)F)(F)F*	1.38	0.138	0.145	Training
13	Poly(octafluoropentyl acrylate)<br><br>*C(=O)(CC)OC(C(C(C(C)(F)F)(F)F)(F)F)(F)F*	1.38	0.140	0.135	Prediction
14	Poly(methyl 3,3,3-trifluoropropyl siloxane)<br><br>*[SiH](O)(CCC(F)(F)F)C*	1.38	0.141	0.148	Training
15	Poly(chlorotrifluoroethylene)<br><br>*C(C(F)Cl)(F)F*	1.39	0.143	0.155	Training
16	Poly(2-(heptafluorobutoxy)ethyl acrylate)<br><br>*C(=O)(CC)OCCOC(C(C(CF)(F)F)(F)F)(F)F*	1.39	0.143	0.144	Training

17	Poly(2,2,3,4,4,4-hexafluorobutyl acrylate)<br><br>*C(=O)(CC)OCC(C(C(F)(F)F)F)(F)F*	1.39	0.144	0.144	Prediction
18	Poly(methyl hydro siloxane)<br><br>*C[SiH2]O*	1.40	0.145	0.161	Training
19	Poly(methacrylic acid), sodium salt<br><br>*C(=O)(C(C)C)O*	1.43	0.155	0.172	Training
20	Poly(dimethyl siloxane)<br><br>*[SiH](O)(C)C*	1.40	0.147	0.158	Training
21	Poly(trifluoroethyl acrylate)<br><br>*C(=O)(CC)OC(CF)(F)F*	1.41	0.148	0.152	Training
22	Poly(2,2,2-trifluoroethyl acrylate)<br><br>*C(=O)(CC)OCC(F)(F)F*	1.41	0.149	0.151	Prediction
23	Poly(2-(1,1,2,2-tetrafluoroethoxy)ethyl acrylate<br><br>*C(=O)(CC)OCCOC(C(F)F)(F)F*	1.41	0.150	0.150	Training
24	Poly(trifIuoroisopropyl methacrylate)<br><br>*C(=O)(CC)OC(C(F)F)(C)F*	1.42	0.152	0.153	Training
25	Poly (2,2,2-trifluoro-1 -methylethyl methacrylate)<br><br>*C(=O)(CC)OC(C(F)(F)F)C*	1.42	0.152	0.152	Training
26	Poly(2-trifluoroethoxyethyl acrylate)<br><br>*C(=O)(CC)OCCOC(CF)(F)F*	1.42	0.152	0.156	Prediction
27	Poly(vinylidene fluoride)<br><br>*C(C)(F)F*	1.42	0.152	0.136	Training
28	Poly(2,2,3,3-tetrafluoropropyl methacrylate)<br><br>*C(=O)(CC)OCC(C(F)F)(F)F*	1.42	0.152	0.151	Training
29	Poly(methyl hexyl siloxane)<br><br>*[SiH](O)(CCCCCC)C*	1.44	0.159	0.171	Prediction
30	Poly(methyl octadecyl siloxane)<br><br>*[SiH](O)(CCCCCCCCCCCCCCCCCC)C*	1.44	0.159	0.171	Training
31	Poly(vinyl isobutyl ether)<br><br>*O(CC(C)C)CC*	1.45	0.162	0.167	Training
32	Poly[oxy(methyl n-hexadecylsilylene)]<br><br>*[SiH](CCCCCCCCCCCCCCCC)(C)O*	1.45	0.162	0.171	Prediction
33	Poly(ethylene oxide)<br><br>*C(C)O*	1.45	0.163	0.164	Training
34	Poly(vinyl ethyl ester)<br><br>*O(C(=O)C)CC*	1.45	0.163	0.164	Training
35	Poly(methyl tetradecyl siloxane)<br><br>*[SiH](O)(CCCCCCCCCCCCCC)C*	1.46	0.163	0.171	Training

36	Poly(ethylene glycol mono-methyl ether)<br><br>*C(CO)OC*	1.46	0.163	0.163	Prediction
37	Poly(oxyethylene)<br><br>*C(CO)O*	1.46	0.163	0.176	Training
38	Poly(vinyl butyl ether)<br><br>*O(CCCC)CC*	1.46	0.163	0.170	Training
39	Poly(propylene oxide)<br><br>*CC(C)O*	1.46	0.163	0.158	Training
40	Poly(3-butoxypropylene oxide)<br><br>*CC(COCCCC)O*	1.46	0.164	0.169	Prediction
41	Poly(methyl vinyl ether)<br><br>*O(CC)C*	1.47	0.167	0.166	Training
42	Poly(vinyl pentyl ether)<br><br>*O(CCCCC)CC*	1.46	0.164	0.173	Training
43	Poly(3-hexoxypropylene oxide)<br><br>*CC(COCCCCCC)O*	1.46	0.164	0.171	Training
44	Poly(hexyl vinyl ether)<br><br>*O(CC)CCCCCC*	1.46	0.164	0.172	Training
45	Poly(4-fluoro-2-trifluoromethylstyrene)<br><br>*CCc1c(cc(cc1)F)C(F)(F)F*	1.46	0.164	0.178	Prediction
46	Poly(octyl vinyl ether)<br><br>*O(CC)CCCCCCCC*	1.46	0.165	0.173	Training
47	Poly(vinyl n-octyl acrylate)<br><br>*CCC(=O)OC(CCCCCCC)CC*	1.46	0.165	0.174	Training
48	Poly(vinyl 2-ethylhexyl ether)<br><br>*O(CC(CCCC)CC)CC*	1.46	0.165	0.173	Training
49	Poly(vinyl decyl ether)<br><br>*O(CCCCCCCCCC)CC*	1.46	0.165	0.173	Prediction
50	Poly(2-methoxyethyl acrylate)<br><br>*C(=O)(CC)OCCOC*	1.46	0.165	0.168	Training
51	Poly(3-methoxypropylene oxide)<br><br>*CC(COC)O*	1.46	0.165	0.160	Training
52	Poly(4-methyl-1-pentene)<br><br>*CCCC(C)C*	1.46	0.165	0.168	Training
53	Poly(acryloxypropyl methyl siloxane)<br><br>*[SiH](O)(C)CCCOC(=O)C=C*	1.46	0.165	0.172	Prediction
54	Poly(ethyl vinyl ether)<br><br>*CCOCC*	1.45	0.161	0.165	Training

55	Poly(t-butyl methacrylate)<br><br>*C(=O)(C(C)C)OC(C)(C)C*	1.46	0.165	0.161	Training
56	Poly(vinyl dodecyl ether)<br><br>*O(CCCCCCCCCCCC)CC*	1.46	0.166	0.174	Training
57	Poly(3-ethoxypropyl acrylate)<br><br>*C(=O)(CC)OCCCOCC*	1.47	0.166	0.170	Training
58	Poly(vinyl propionate)<br><br>*C(=O)(CC)OCC*	1.47	0.166	0.171	Prediction
59	Polyoxyoctamethylene<br><br>*C(CCCCCCC)O*	1.47	0.167	0.177	Training
60	Cellulose acetate propionate<br><br>*O1[C@@H](C[C@@H]([C@H]([C@@H]1O[C@H]1[C@@H]([C@H]([C@@H](O[C@@H]1COC(=O)CC)O)OC(=O)CC)OC(=O)CC)OC(=O)CC)OC(=O)CC)COC(=O)CC*	1.47	0.167	0.173	Training
61	Poly(1-octadecene)<br><br>*CCCCCCCCCCCCCCCCCC*	1.47	0.168	0.176	Prediction
62	Poly(2-ethoxyethyl acrylate)<br><br>*C(=O)(CC)OCCOCC*	1.47	0.168	0.170	Training
63	Poly(3-methoxypropyl acrylate)<br><br>*C(=O)(CC)OCCCOC*	1.47	0.168	0.171	Training
64	Poly(isopropyl acrylate)<br><br>*C(=O)(CC)OC(C)C*	1.47	0.168	0.167	Training
65	Poly(1-decene)<br><br>*CCCCCCCCCC*	1.47	0.168	0.177	Prediction
66	Poly(propylene)<br><br>*CCC*	1.47	0.168	0.165	Training
67	Poly(dodecyl methacrylate)<br><br>*C(=O)(C(C)C)OCCCCCCCCCCCC*	1.47	0.168	0.173	Training
68	Poly(vinyl sec-butyl ether) (isotactic)<br><br>*O(C(CC)C)CC*	1.47	0.168	0.169	Training
69	Poly(oxyethyleneoxysuccinoyl) (poly(ethylene succinate))<br><br>*C(=O)(CCC=O)OCCO*	1.47	0.169	0.178	Prediction
70	Poly(tetradecyl acrylate)<br><br>*C(=O)(CC)OCCCCCCCCCCCCCC*	1.47	0.169	0.175	Training
71	Poly(tetradecyl methacrylate)<br><br>*C(=O)(C(C)C)OCCCCCCCCCCCCCC*	1.47	0.169	0.173	Training
72	Poly(hexadecyl acrylate)<br><br>*C(=O)(CC)OCCCCCCCCCCCCCCCC*	1.48	0.169	0.175	Training
73	Poly(hexadecyl methacrylate)<br><br>*C(=O)(C(C)C)OCCCCCCCCCCCCCCCC*	1.48	0.169	0.173	Prediction

74	Poly(vinyl formate)<br><br>*C(OC=O)C*	1.48	0.169	0.170	Training
75	Ethylene/vinyl acetate copolymer-40% vinyl acetate<br><br>*CCCCOC(=O)C*	1.48	0.169	0.169	Training
76	Poly(ethylene)<br><br>*CC*	1.51	0.179	0.172	Training
77	Poly(2-fluoroethyl methacrylate)<br><br>*C(=O)(C(C)C)OCCF*	1.48	0.169	0.167	Training
78	Poly(octyl methyl silane)<br><br>*[SiH2](C)CCCCCCCC*	1.48	0.170	0.174	Prediction
79	Poly(methyl acrylate)<br><br>*C(=O)(OC)CC*	1.48	0.170	0.173	Training
80	Poly(dicyanopropyl siloxane)<br><br>*[SiH](CCCC#N)(CCCC#N)O*	1.48	0.170	0.174	Training
81	Poly(dimethylsiloxane-co-alpha-methyl styrene)<br><br>*c1ccccc1C(C[SiH](O[Si](C)(C)C)C)C*	1.48	0.170	0.176	Training
82	Poly(ethylene-co-propylene) (EPR-rubber)<br><br>*CC(CC)C*	1.48	0.170	0.170	Prediction
83	Poly(isobutyl methacrylate)<br><br>*C(=O)(C(C)C)OCC(C)C*	1.48	0.170	0.168	Training
84	Poly(oxymethylene)<br><br>*CO*	1.48	0.170	0.166	Training
85	Poly(sec-butyl methacrylate)<br><br>*C(=O)(C(C)C)OC(CC)C*	1.48	0.170	0.169	Training
86	Poly(hexyl methacrylate)<br><br>*C(=O)(C(C)C)OCCCCCC*	1.48	0.171	0.172	Training
87	Poly(butyl methacrylate)<br><br>*C(=O)(CC)OCCCC*	1.48	0.171	0.174	Training
88	Poly(ethylidene dimethacrylate)<br><br>*C(C)(OC(=O)C(=C)C)OC(=O)CC*	1.48	0.171	0.171	Training
89	Poly(2-ethoxyethyl methacrylate)<br><br>*C(=O)(C(C)C)OCCOCC*	1.48	0.171	0.168	Prediction
90	Poly(ethylene maleate)<br><br>*C(=O)(C(CC(=O)O)CC)O*	1.48	0.171	0.178	Training
91	Poly(propyl methacrylate)<br><br>*C(=O)(C(C)C)OCCC*	1.48	0.171	0.171	Training
92	Poly(3,3,5-trimethylcyclohexyl methacrylate)<br><br>*C(=O)(C(C)C)OC1CC(CC(C1)C)(C)C*	1.49	0.172	0.173	Training

93	Poly(ethyl methacrylate)<br><br>*C(=O)(C(C)C)OCC*	1.49	0.172	0.168	Prediction
94	Poly(vinyl butyral)<br><br>*C1(OC(OC(C1)CC)CCC)C*	1.49	0.172	0.177	Training
95	Poly(2-nitro-2-methylpropyl methacrylate)<br><br>*C(=O)(C(C)C)OCC(C)(C)[N+](=O)[O-]*	1.49	0.172	0.166	Training
96	Poly(dimethylsiloxane-co-diphenylsiloxane)<br><br>*c1ccccc1[Si](O[Si](O[Si](c1ccccc1)(O)c1ccccc1)(C)C)(O)c1ccccc1*	1.48	0.170	0.190	Prediction
97	Poly(1,1-diethylpropyl methacrylate)<br><br>*C(=O)(C(C)C)OC(CC)(CC)CC*	1.49	0.173	0.173	Training
98	Poly(methyl methacrylate)<br><br>*C(=O)(C(C)C)OC*	1.49	0.173	0.169	Prediction
99	Poly(2-decyl-1,3-butadiene)<br><br>*C=C(CC)CCCCCCCCCC*	1.49	0.173	0.179	Training
100	Poly(2-decyl-1,4-butadiene)<br><br>*C(CC)C(=C)CCCCCCCCC*	1.49	0.173	0.177	Training
101	Poly(mercaptopropyl methyl siloxane)<br><br>*[Si@@H](O[SiH3])(C)CCCS*	1.49	0.173	0.173	Training
102	Poly(ethyl glycolate methacrylate)<br><br>*C(=O)(COC(=O)C(C)C)OCC*	1.49	0.173	0.168	Prediction
103	Poly(3-methylcyclohexyl methacrylate)<br><br>*C(=O)(C(C)C)OC1CC(CCC1)C*	1.49	0.175	0.179	Training
104	Poly(cyclohexyl alpha-ethoxyacrylate)<br><br>*CC(C(=O)OC1CCCCC1)OCC*	1.50	0.175	0.179	Training
105	Poly(1-butene)<br><br>*CCCC*	1.51	0.179	0.176	Training
106	Poly(4-methylcyclohexyl methacrylate)<br><br>*C(=O)(C(C)C)OC1CCC(CC1)C*	1.50	0.175	0.181	Training
107	Poly(decamethylene glycol dimethacrylate)<br><br>*C(OCOCOCOCOCOCOCOCOCOC(=O)C(C)C)OC(=O)C(=C)C*	1.50	0.176	0.159	Prediction
108	Poly (1,2-butadiene)<br><br>*CCC=C*	1.50	0.176	0.187	Training
109	Poly(2-bromo-4-trifluoromethyl styrene)<br><br>*CCc1c(cc(cc1)C(F)(F)F)Br*	1.50	0.176	0.192	Training
110	Poly(2-heptyl-1,3-butadiene)<br><br>*C=C(CC)CCCCCCC*	1.50	0.176	0.179	Training
111	Poly(2-heptyl-1,4-butadiene)<br><br>*CCCC(=C)CCCCCC*	1.50	0.176	0.179	Prediction

112	Poly(sec-butyl alpha-chloroacrylate)<br><br>*C(=O)(C(C)Cl)OC(CC)C*	1.50	0.176	0.179	Training
113	Poly(vinyl formal)<br><br>*C1COCOC1C*	1.50	0.176	0.182	Training
114	Poly(vinyl methyl ketone)<br><br>*C(=O)(C)CC*	1.50	0.176	0.175	Training
115	Poly(2-isopropyl-1,4-butadiene)<br><br>*C/C(=C/C)/C(C)C*	1.50	0.177	0.178	Prediction
116	Poly(ethyl alpha-chloroacrylate)<br><br>*C(=O)(C(C)Cl)OCC*	1.50	0.177	0.181	Training
117	Poly(2-isopropyl-1,3-butadiene)<br><br>*C=C(CC)C(C)C*	1.50	0.177	0.178	Training
118	Poly(2-methylcyclohexyl methacrylate)<br><br>*C(=O)(C([CH])C)OC1C(CCCC1)C*	1.50	0.177	0.181	Training
119	Poly(1,1-dimethylethylene)<br><br>*C(C)(C)C*	1.51	0.178	0.158	Prediction
120	Poly(2-tert-butyl-1,3-butadiene)<br><br>*C=C(CC)C(C)(C)C*	1.51	0.178	0.172	Training
121	Poly(bornyl methacrylate)<br><br>*C(=O)(C(C)C)OC1CC2CCC1(C2(C)C)C*	1.51	0.178	0.183	Training
122	Poly(2-t-butyl-1,4-butadiene)<br><br>*C/C(=C/C)/C(C)(C)C*	1.51	0.178	0.172	Training
123	Poly(ethylene glycol dimethacrylate)<br><br>*C(=O)(OCCOC(=O)C(=C)C)C(=C)C*	1.51	0.178	0.176	Prediction
124	Poly(cyclohexyl methacrylate)<br><br>*C(=O)(C(C)C)OC1CCCCC1*	1.51	0.178	0.182	Training
125	Poly(cyclohexanediol-1,4-dimethacrylate)<br><br>*C1(CCC(CC1)(/C=C(/C(=O)O)\C)O)(O)CC(C(=O)O)C*	1.51	0.178	0.181	Training
126	Poly(acrylic acid)<br><br>*C(=O)(CC)O*	1.53	0.185	0.177	Training
127	Gutta percha b<br><br>*C/C(=C/CC/C(=C/C)/C)/C*	1.51	0.179	0.179	Training
128	Poly(tetrahydrofurfuryl methacrylate)<br><br>*C(=O)(C(C)C)OCC1CCCO1*	1.51	0.179	0.179	Prediction
129	Natural rubber<br><br>*C/C(=C/C)/C*	1.52	0.182	0.178	Training
130	Polyacetal<br><br>*CC(OCC)OCC*	1.51	0.179	0.161	Training

131	Shellac<br><br>*C1(CCC2C31CC(C2(C)C)C(=CC3CC(=O)CCCCCCCCC(O)CCCCCCO)C(=O)O)C*	1.51	0.179	0.183	Prediction
132	Poly(1-methylcyclohexyl methacrylate)<br><br>*C(=O)(C(C)C)OC1(CCCCC1)C*	1.51	0.179	0.178	Training
133	Poly(2-hydroxyethyl methacrylate)<br><br>*C(=O)(C(C)C)OCCO*	1.51	0.180	0.171	Training
134	Poly(vinyl methacrylate)<br><br>*CC(C)C(=O)OC=C*	1.51	0.180	0.174	Training
135	Poly(vinyl chloroacetate)<br><br>*C(=O)(CCl)OCC*	1.51	0.180	0.186	Prediction
136	Poly(N-butyl methacrylamide)<br><br>*C(=O)(C(C)C)NCCCC*	1.51	0.180	0.171	Training
137	Terpene resin<br><br>*C1CC(CC=C1C)C(C)C*	1.51	0.180	0.187	Training
138	Poly(2-chloroethyl methacrylate)<br><br>*C(=O)(C(C)C)OCCCl*	1.52	0.181	0.182	Training
139	Poly(methyl alpha-chloroacrylate)<br><br>*C(=O)(C(C)Cl)OC*	1.52	0.181	0.184	Prediction
140	Poly(2-diethylaminoethyl methacrylate)<br><br>*C(=O)(C(C)C)OCCN(CC)CC*	1.52	0.181	0.168	Training
141	Poly(2-chlorocyclohexyl methacrylate)<br><br>*C(=O)(C(C)C)OC1C(CCCC1)Cl*	1.52	0.181	0.188	Training
142	Poly(acrylonitrile)<br><br>*C(#N)CC*	1.52	0.181	0.190	Training
143	Poly(allyl methacrylate)<br><br>*C(=O)(C(=C)C)OCCC*	1.52	0.182	0.177	Prediction
144	Poly(butadiene-co-acrylonitrile)<br><br>*C(#N)C(C)C/C=C/C*	1.52	0.182	0.188	Training
145	Poly(methacrylonitrile)<br><br>*C(#N)C(C)C*	1.52	0.182	0.181	Training
146	Poly(methyl isopropenyl ketone)<br><br>*C(=O)(C(C)C)C*	1.52	0.182	0.170	Prediction
147	Poly((N-2-methoxyethyl)methacrylamide)<br><br>*C(=O)(C(C)C)NCCOC*	1.52	0.183	0.166	Training
148	Poly(2,3-dimethylbutadiene) [methyl rubber]<br><br>*C/C(=C(/CC/C(=C(/C)\C)/C)\C)/C*	1.53	0.183	0.177	Training
149	Poly(ω-dodecanamide), Nylon 12<br><br>*C(=O)CCCCCCCCCCCN*	1.53	0.183	0.178	Training

150	Poly(1,3-dichloropropyl methacrylate)<br><br>*C(=O)(C(C)C)OC(CCCl)Cl*	1.53	0.184	0.184	Prediction
151	Poly(2-chloro-1-(chloromethyl)ethyl methacrylate)<br><br>*C(=O)(C(C)C)OC(CCl)CCl*	1.53	0.184	0.188	Training
152	Poly(acrolein)<br><br>*C(=O)C=C*	1.53	0.184	0.198	Training
153	Poly(N-methyl-methacrylamide)<br><br>*C(=O)(C(C)C)NC*	1.54	0.188	0.170	Training
154	Nylon 6 [Poly(caprolactam)]<br><br>*NCCCCCC=O*	1.53	0.185	0.181	Prediction
155	Poly(1-vinyl-2-pyrrolidone)<br><br>*N1(C(=O)CCC1)CC*	1.53	0.185	0.186	Training
156	Poly(hexamethylene sebacamide)<br><br>*NCCCCCCNC(=O)CCCCCCCCC(=O)NCCCCCCNC(=O)*<br><br>*CCCCCCCCC(=O)NCCCCCCNC(=O)CCCCCCCCC=O*	1.53	0.185	0.174	Training
157	Poly(iminoadipoyliminohexamethylene) (nylon 6,6)<br><br>*C(=O)CCCCC(=O)NCCCCCCN*	1.53	0.185	0.177	Training
158	Poly(N-methylmaleimide-alt-isobutene)<br><br>*CC(C1C(=O)N(C(=O)C1)C)(C)C*	1.53	0.185	0.177	Prediction
159	Poly(cyclohexyl alpha-chloroacrylate)<br><br>*C(=O)(C(C)Cl)OC1CCCCC1*	1.53	0.185	0.190	Training
160	Poly(2-chloroethyl alpha-chloroacrylate)<br><br>*C(=O)(C(C)Cl)OCCCl*	1.53	0.186	0.191	Training
161	Poly(methyl phenyl siloxane)<br><br>*[SiH](O)(c1ccccc1)C*	1.54	0.188	0.191	Training
162	Poly(butadiene-co-styrene)<br><br>*C/C=C/CCCc1ccccc1*	1.54	0.186	0.202	Training
163	Poly(2-aminoethyl methacrylate)<br><br>*C(=O)(C(C)C)OCCN*	1.54	0.187	0.170	Prediction
164	Poly(furfuryl methacrylate)<br><br>*C(=O)(C(C)C)OCc1ccco1*	1.54	0.187	0.188	Training
165	Poly(butylmercaptyl methacrylate)<br><br>*CC(C(=O)OCCCCS)C*	1.54	0.187	0.182	Training
166	Poly(vinyl chloride)<br><br>*C(C)Cl*	1.54	0.187	0.190	Training
167	Poly(1-phenyl-n-amyl methacrylate)<br><br>*C(=O)(C(C)C)OC(CCCC)c1ccccc1*	1.54	0.187	0.190	Prediction
168	Poly(cyclohexyl alpha-bromoacrylate)	1.54	0.188	0.197	Training

	C(=O)(C(C)Br)OC1CCCCC1				
169	Poly(sec-butyl alpha-bromoacrylate)<br>*C(=O)(C(C)Br)OC(CC)C*	1.54	0.188	0.188	Prediction
170	Poly(2-bromoethyl methacrylate)<br>*C(=O)(C(C)C)OCCBr*	1.54	0.188	0.192	Training
171	Poly(dihydroabietic acid)<br>*C(=O)([C@@]1(C)[C@H]2[C@@]([C@H]3CCC(C(C)C)CC3=CC2)(CCC1)C)O*	1.54	0.189	0.189	Training
172	Poly(abietic acid)<br>*C(=O)([C@@]1(C)[C@H]2[C@@]([C@H]3CCC(=CC3=CC2)C(C)C)(CCC1)C)O*	1.55	0.189	0.191	Training
173	Poly(ethylmercaptyl methacrylate)<br>*CC(C(=O)OCCS)C*	1.55	0.189	0.184	Prediction
174	Poly(N-allyl methacrylamide)<br>*C(=O)(CC)NCC*	1.55	0.190	0.172	Training
175	Poly(1-phenylethyl methacrylate)<br>*C(=O)(C(C)C)OC(C)c1ccccc1*	1.55	0.190	0.191	Training
176	Poly(2-vinyltetrahydrofuran)<br>*O1C(CCC1)CC*	1.55	0.190	0.189	Training
177	Poly(methyl m-chlorophenylethyl siloxane)<br>*[SiH](O)(CCc1cc(ccc1)Cl)C*	1.55	0.190	0.186	Prediction
178	Poly(vinylfuran)<br>*o1c(ccc1)CC*	1.55	0.190	0.203	Training
179	Poly[oxy(methyl m-chlorophenylethylsilylene)]<br>*[SiH](C(C)c1cc(ccc1)Cl)(C)O*	1.55	0.190	0.186	Training
180	Urea-formaldehyde resin<br>*N(C(=O)N)CO*	1.55	0.190	Not Predicted	Not Included
181	Poly(isopropyl methacrylate)<br>*C(=O)(C(C)C)OC(C)C*	1.55	0.191	Not Predicted	Not Included
182	Poly(p-methoxybenzyl methacrylate)<br>*C(=O)(C(C)C)OCc1ccc(cc1)OC*	1.55	0.191	0.189	Training
183	Poly(p-isopropyl styrene)<br>*CCc1ccc(cc1)C(C)C*	1.55	0.191	0.196	Prediction
184	Poly(p,p-xylylenyl dimethacrylate)<br>*c1(ccc(cc1)COC(=O)C(C)C)COC(=O)C(C)C*	1.56	0.192	0.184	Training
185	Poly(cyclohexyl methyl silane)<br>*[SiH2](C)C1CCCCC1*	1.56	0.192	0.188	Training
186	Poly(1-phenylallyl methacrylate)<br>*C(=O)(CC)OC(C)c1ccccc1*	1.56	0.192	0.195	Training
187	Poly(p-cyclohexylphenyl methacrylate)<br>*C(=O)(C(C)C)Oc1ccc(cc1)C1CCCCC1*	1.56	0.192	0.194	Prediction

188	Poly(chloroprene)<br><br>*C(=C\C)(\Cl)/C*	1.56	0.193	0.193	Training
189	Poly(2-phenylethyl methacrylate)<br><br>*C(=O)(C(C)C)OCCc1ccccc1*	1.56	0.193	0.192	Training
190	Poly(methyl m-chlorophenyl siloxane)<br><br>*[SiH](O[SiH3])(c1cccc(c1)Cl)C*	1.56	0.193	0.182	Training
191	Poly [4,4-heptane bis(4-phenyl)carbonate]<br><br>*OC(=O)OCCCCCCCOc1ccc(cc1)c1ccc(cc1)OCCCCCCCOC(=O)O*	1.56	0.193	0.185	Prediction
192	Poly(oxycarbonyloxy-1,4-phenylene-1-propylbutylidene-1,4-phenylene)<br><br>*c1(c(cccc1)C(CCC)CCC)c1ccc(cc1)OC(=O)O*	1.56	0.193	0.196	Training
193	Poly(1-(o-chlorophenyl)ethyl methacrylate)<br><br>*C(=O)(C(C)C)OC(C)c1c(cccc1)Cl*	1.56	0.194	0.193	Training
194	Poly(styrene-co-maleic anhydride)<br><br>*CC(C1C(=O)OC(=O)C1)c1ccccc1*	1.56	0.194	0.203	Training
195	Styrene/maleic anhydride copolymer<br><br>*C1(=O)OC(=O)CC1CCc1ccccc1*	1.56	0.194	0.203	Prediction
196	Poly(1-phenylcyclohexyl methacrylate)<br><br>*C(=O)(C(C)C)OC1(CCCCC1)c1ccccc1*	1.56	0.194	0.195	Training
197	Poly(2,2,2'-trimethylhexamethylene terephthalamide)<br><br>*C(=O)c1ccc(cc1)C(=O)NCC(CC(CCN)C)(C)C*	1.57	0.195	0.185	Training
198	Poly(methyl α-bromoacrylate)<br><br>*C(=O)(C(C)Br)OC*	1.57	0.195	0.195	Training
199	Poly(oxycarbonyloxy-1,4-phenylene-1,3-dimethylbutylidene-1,4-phenylene)<br><br>*c1(c(cccc1)C(CC(C)C)C)c1ccc(cc1)OC(=O)O*	1.57	0.195	0.195	Prediction
200	Poly(benzyl methacrylate)<br><br>*C(=O)(C(C)C)OCc1ccccc1*	1.57	0.195	0.195	Training
201	Poly(2-(phenylsulfonyl)ethyl methacrylate)<br><br>*C(=O)(C(C)C)OCCS(=O)(=O)c1ccccc1*	1.57	0.195	0.185	Training
202	Poly(m-cresyl methacrylate)<br><br>*c1(cc(ccc1)C)OC(=O)C(C)C*	1.57	0.195	0.190	Training
203	Poly(oxycarbonyloxy-1,4-phenyleneisobutylidene-1,4-phenylene)<br><br>*c1(c(cccc1)CC(C)C)c1ccc(cc1)OC=O*	1.57	0.196	0.201	Prediction
204	Poly[1,1-(2-methyl propane) bis(4-phenyl)carbonate]<br><br>*O(C(=O)Oc1ccccc1)c1ccc(cc1)C(C(C)C)c1cccc(OC(=O)Oc2ccccc2)c1*	1.57	0.196	0.200	Training
205	Poly(o-methoxyphenol methacrylate)<br><br>*c1(c(cccc1)OC)OC(=O)C(C)C*	1.57	0.196	0.189	Training
206	Poly(phenyl methacrylate)<br><br>*C(=O)(C(C)C)Oc1ccccc1*	1.57	0.196	0.195	Training

207	Poly(o-cresyl methacrylate)<br><br>*c1(c(cccc1)C)OC(=O)C(C)C*	1.57	0.196	0.192	Prediction
208	Poly(diallyl phthalate)<br><br>*C(=O)(c1c(C(=O)OCCC)cccc1)OCCC*	1.57	0.196	0.190	Training
209	Poly(2,3-dibromopropyl methacrylate)<br><br>*C(=O)(C(C)C)OCC(CBr)Br*	1.57	0.197	0.198	Training
210	Poly(oxycarbonyloxy-1,4-phenylene-1-methyl-butylidene-1,4-phylene)<br><br>*c1(c(cccc1)C(CCC)C)c1ccc(cc1)OC(=O)O*	1.57	0.197	0.198	Training
211	Poly(2,6-dimethyl-p-phenylene oxide)<br><br>*c1c(c(c(cc1)C)O)C*	1.58	0.197	0.203	Prediction
212	Poly(ethylene terephthalate)<br><br>*C(=O)(c1ccc(C(=O)O)cc1)OCC*	1.58	0.197	0.197	Training
213	Poly(vinyl benzoate)<br><br>*C(=O)(c1ccccc1)OCC*	1.58	0.198	0.200	Training
214	Poly(4-methylstyrene)<br><br>*CCc1ccc(cc1)C*	1.58	0.198	0.202	Training
215	Poly(oxycarbonyloxy-1,4-phenylenebutylidene-1,4-phenylene)<br><br>*c1(c(cccc1)CCCC)c1ccc(cc1)OC=O*	1.58	0.198	0.204	Prediction
216	Poly(1,2-diphenylethyl methacrylate)<br><br>*C(=O)(C(C)C)OC(Cc1ccccc1)c1ccccc1*	1.58	0.199	0.200	Training
217	Poly(o-chlorobenzyl methacrylate)<br><br>*C(=O)(C(C)C)OCc1c(cccc1)Cl*	1.58	0.199	0.197	Training
218	Poly(Bisphenol B carbonate)<br><br>*c1cc(ccc1O)C(C)(CC)c1ccc(cc1)OC=O*	1.58	0.199	0.201	Training
219	Poly(oxypentaerythritoloxyphthaloyl)<br><br>*C(=O)c1c(C=O)c(c(cc1)O)OCC(CO)(CO)CO*	1.58	0.200	0.193	Prediction
220	Poly(m-nitrobenzyl methacrylate)<br><br>*C(=O)(C(C)C)OCc1cc(ccc1)[N+](=O)[O-]*	1.58	0.200	0.190	Training
221	Poly(2-methoxystyrene)<br><br>*CCc1c(cccc1)OC*	1.59	0.200	0.200	Training
222	Poly(oxycarbonyloxy-1,4-phenyleneisopropylidene-1,4-phenylene)<br><br>*c1(ccccc1)C(C)(C)c1ccc(cc1)OC(=O)O*	1.59	0.200	0.201	Training
223	Poly(N-(2-phenylethyl)methacrylamide)<br><br>*C(=O)(C(C)C)NCCc1ccccc1*	1.59	0.200	0.192	Prediction
224	Bisphenol-A polycarbonate<br><br>*c1cc(ccc1O)C(C)(C)c1ccc(cc1)OC=O*	1.59	0.200	0.201	Training
225	Poly(Bisphenol A carbonate), PC<br><br>*Oc1ccc(cc1)C(C)(C)c1ccc(cc1)OC(=O)O*	1.59	0.200	0.199	Training

226	Poly(4-methoxy-2-methylstyrene)<br><br>*CCc1c(cc(cc1)OC)C*	1.59	0.201	0.195	Training
227	Poly(2-methylstyrene)<br><br>*CCc1c(cccc1)C*	1.59	0.201	0.203	Prediction
228	Polystyrene<br><br>*CCc1ccccc1*	1.59	0.201	0.211	Training
229	Poly(oxycarbonyloxy-1,4-phenylenecyclohexylidene-1,4-phenylene)<br><br>*c1(ccccc1)C1(CCCCC1)c1ccc(cc1)OC(=O)O*	1.59	0.201	0.205	Training
230	Poly(diphenylmethyl methacrylate)<br><br>*C(=O)(C(C)C)OC(c1ccccc1)c1ccccc1*	1.59	0.202	0.203	Training
231	Poly(oxycarbonyloxy-1,4-phenyleneethylidene-1,4-phenylene)<br><br>*c1(ccccc1)C(C)c1ccc(cc1)OC(=O)O*	1.59	0.202	0.205	Prediction
232	Poly(2,5-dimethyl-1,4-phenylene ethylene)<br><br>*C(C)c1cc(ccc1C)C*	1.60	0.203	0.199	Training
233	Poly(4-bromophenyl methacrylate)<br><br>*C(=O)(C(C)C)Oc1ccc(cc1)Br*	1.60	0.203	0.205	Training
234	Poly(propylene sulfide)<br><br>*CCCS*	1.60	0.203	0.195	Training
235	Poly(N-benzyl methacrylamide)<br><br>*C(=O)(C(C)C)NCc1ccccc1*	1.60	0.203	0.195	Prediction
236	Poly(4-methoxystyrene)<br><br>*CCc1ccc(cc1)OC*	1.60	0.203	0.200	Training
237	Poly(2,6,3',5'-tetrachloro bisphenol A carbonate)<br><br>*Oc1cc(c(c(c1)Cl)C(C)(C)c1cc(c(c(c1)Cl)OC(=O)O)Cl)Cl*	1.60	0.204	0.204	Training
238	Hard Rubber (32% S)<br><br>*C=C(C)C(CC)SC(C(=C)C)CC*	1.60	0.204	0.185	Training
239	Poly(vinylidene chloride)<br><br>*C(=C)(Cl)Cl*	1.60	0.204	0.202	Prediction
240	Poly(o-chlorodiphenylmethyl methacrylate)<br><br>*C(=O)(C(=C)C)OC(c1c(cccc1)Cl)c1ccccc1*	1.60	0.205	0.207	Training
241	Poly(oxycarbonyloxy-1,4-(2,6-dichloro)phenylene-isopropylidene-1,4-(2,6-dichloro)phenylene)<br><br>*c1(cc(cc(c1)Cl)Cl)C(C)(C)c1cc(c(c(c1)Cl)OC(=O)O)Cl*	1.61	0.206	0.206	Training
242	Poly(oxycarbonyloxybis(1,4-(3,5-dichlorophenylene)))<br><br>*c1(c2c(cc(cc2Cl)OC(=O)O)Cl)cc(cc(c1)Cl)Cl*	1.61	0.206	0.212	Training
243	Poly(2-chlorostyrene)<br><br>*C=Cc1c(cccc1)Cl*	1.61	0.207	0.218	Prediction
244	Poly(alpha-methylstyrene)<br><br>*CC(c1ccccc1)C*	1.61	0.207	0.202	Training

245	Poly(phenyl alpha-bromoacrylate)<br>*C(=O)(C(C)Br)Oc1ccccc1*	1.61	0.207	0.208	Training
246	Poly(p-divinylbenzene)<br>*c1(ccc(cc1)CC)CC*	1.62	0.208	0.202	Training
247	PoIy(N-vinyl phthalimide)<br>*C1(=O)c2c(C(=O)N1CC)cccc2*	1.62	0.210	0.206	Prediction
248	Poly(2,6-dichlorostyrene)<br>*CCc1c(cccc1Cl)Cl*	1.62	0.211	0.212	Training
249	Poly(chloro-p-xylylene)<br>*c1cc(c(cc1C)Cl)C*	1.63	0.212	0.208	Training
250	Poly(beta-naphthyl methacrylate)<br>*C(=O)(C(C)C)Oc1cc2c(cc1)cccc2*	1.63	0.212	0.203	Training
251	Poly(alpha-naphthyl carbinyl methacrylate)<br>*C(=O)(C(Cc1cccc2ccccc12)C)O*	1.63	0.212	0.207	Prediction
252	Poly(phenyl methyl silane)<br>*[SiH2](C)c1ccccc1*	1.63	0.212	0.196	Training
253	Poly(sulfone)<br>*Oc1ccc(cc1)C(C)(C)c1ccc(cc1)Oc1ccc(cc1)S(=O)(=O)c1ccccc1*	1.63	0.213	0.203	Training
254	Poly(2-vinylthiophene)<br>*c1(cccs1)CC*	1.64	0.214	0.219	Training
255	Poly (2,6-diphenyl-1,4-phenylene oxide)<br>*c1(c(cc(cc1c1ccccc1)O)c1ccccc1)C*	1.64	0.215	0.216	Prediction
256	Poly[oxy(2,6-diphenyI-1,4-phenylene)]<br>*c1c(c(c(cc1)c1ccccc1)O)c1ccccc1*	1.64	0.215	0.219	Training
257	Poly(alpha-naphthyl methacrylate)<br>*C(=O)(C(C)C)Oc1cccc2ccccc12*	1.64	0.215	0.204	Training
258	Poly(p-phenylene ether-sulphone)<br>*c1cc(ccc1)Oc1ccc(cc1)S(=O)=O*	1.65	0.217	0.201	Training
259	Poly(oxycarbonyloxy-1,4-phenylenediphenyl-methylene-1,4-phenylene)<br>*c1(c(cccc1)C(c1ccccc1)c1ccccc1)c1ccc(cc1)OC(=O)O*	1.65	0.219	0.213	Prediction
260	Poly(styrene sulfide)<br>*C(c1ccccc1)CS*	1.66	0.219	0.211	Training
261	Poly(vinyl phenyl sulfide)<br>*S(c1ccccc1)CC*	1.66	0.219	0.214	Training
262	Poly(p-xylene)<br>Cc1ccc(cc1)C	1.67	0.222	0.206	Training
263	Poly(p-xylylene)<br>c1(cccc(c1)Cc1ccccc1)Cc1ccccc1	1.67	0.222	0.214	Prediction

264	Poly(2-vinylnaphthalene)<br><br>c1c(ccc2ccccc12)CC	1.68	0.226	0.217	Training
265	Poly(vinylnaphthalene)<br><br>c1(cccc2ccccc12)CC	1.68	0.226	0.217	Training
266	Poly(N-vinyl carbazole)<br><br>n1(c2ccccc2c2ccccc12)CC	1.68	0.226	0.215	Training
267	Naphthalene-formaldehyde rubber<br><br>c1c(c2c(cc1)cccc2)CO	1.70	0.229	0.220	Prediction
268	Poly(sulfides) (Thiokol)<br><br>CCS(=S)S=S	1.65	0.217	0.225	Training
269	Poly(pentabromophenyl methacrylate)<br><br>C(=O)(C(C)C)Oc1c(c(c(c(c1Br)Br)Br)Br)Br	1.71	0.233	Not Predicted	Not Included
